# Recurrent power outages and the mental health burden of grid reliability in Maryland

**DOI:** 10.1016/j.isci.2026.117513

**Published:** 2026-09-16

**Authors:** Meng Feng, Yueming (Lucy) Qiu, Jie Chen, Jiehong Lou, Yi David Wang

**Affiliations:** 1 School of Public Policy, University of Maryland, College Park, MD 20742, USA; 2 Department of Health Policy and Management, School of Public Health, University of Maryland, College Park, MD 20742, USA; 3 Department of Economics, Virginia Polytechnic Institute and State University, Blacksburg, VA 24061, USA

**Keywords:** power outages, grid reliability, mental health, environmental health, energy infrastructure

## Abstract

Power outages are increasingly shaped by aging infrastructure, climate stress, and rising electricity demand. Their mental health consequences remain poorly quantified, particularly for routine outages outside major disasters. Here, we link 15-min outage records with Maryland Healthcare Cost and Utilization Project State Inpatient Database records from 2018 to 2023 and estimate ZIP-quarter associations with hospitalizations for primary mental health diagnoses. Higher outage exposure was consistently associated with increased hospitalization rates in Poisson pseudo-maximum likelihood models with ZIP code and year-quarter fixed effects and controls for weather, socioeconomic conditions, demographics, healthcare access, and comorbidity. The baseline incidence rate ratio was approximately 1.017, equivalent to a 1.7% increase per one-unit increase in logged outage episodes. Results were robust to alternative thresholds and concentrated among frequent 1–3h disruptions. Instrumental-variable analyses based on grid infrastructure yielded larger positive estimates. These findings suggest that grid reliability is a climate-adaptation and health-system continuity issue.

## Highlights

• Recurrent outages are linked to higher mental health hospitalization rates
• Frequent 1-3 h outages show the strongest and most precise associations
• Grid-infrastructure instruments support a causal outage effect
• Grid reliability is a public health and climate-adaptation priority

## Graphical abstract

**Figure:**
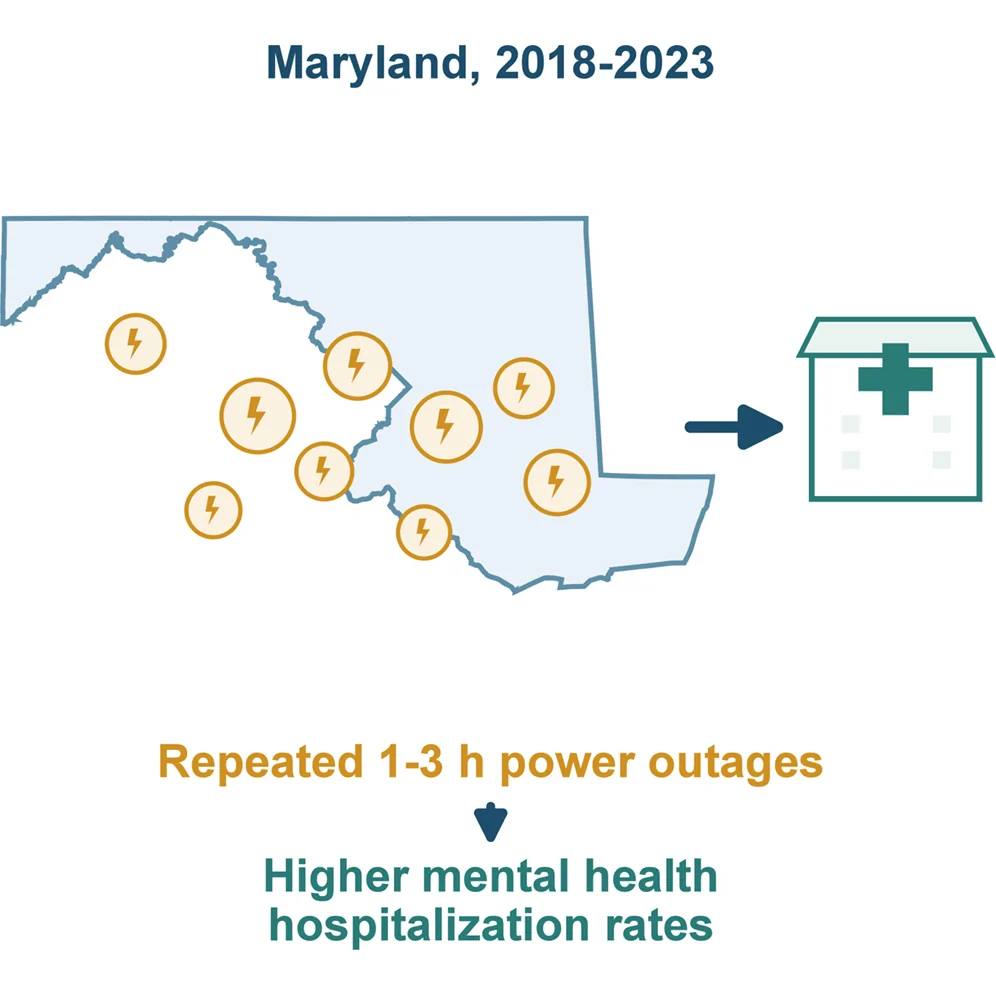

## Teaser

Health sciences; Public health; Mental state; Energy resources; Energy policy; Energy sustainability

## Introduction

Power outages are an increasingly pervasive and intensifying threat to public health in the United States,^1^^,^^2^^,^^3^ yet their impact on mental health remains insufficiently understood. In states such as Maryland, aging grid infrastructure poses a growing risk of power outages, while climate-driven extreme heat may further exacerbate this vulnerability by raising cooling demand, stressing grid performance, and worsening existing energy affordability burdens for low-income households.^4^^,^^5^ Power outages disrupt mental healthcare delivery through multiple, interrelated pathways. Telehealth services may become unavailable, transportation to in-person appointments can be impaired, and pharmacy closures may hinder timely medication refills. During widespread outages, mental health crisis hotlines are often inaccessible when demand surges,^6^^,^^7^ while emergency psychiatric services face increased strain due to system-wide overload. Such mental health shocks may manifest as stress reactions, anxiety, exacerbation of psychotic disorders, or substance-use crises, yet most existing studies rely on survey methods to identify mental health issues without looking at actual clinical data.

Power outages could be life-threatening, particularly for individuals who depend on electricity for medical purposes.^8^ Climate change is expected to increase the frequency, intensity, and duration of extreme weather events, which—compounded by aging infrastructure—may result in more severe and prolonged power disruptions.^9^ As the frequency and duration of power outages continue to rise, medically vulnerable populations—particularly users of electricity-dependent durable medical equipment—are increasingly at risk, with disparities observed across socioeconomic and racial lines.^10^ In 2012, an estimated 685,000 individuals in the US relied on electricity-dependent medical devices while living at home.^8^ Using a comparable Medicare-based measure from the US. Department of Health and Human Services (HHS) empower Program, beneficiaries with claims for electricity-dependent durable medical equipment identified about 3.0 million beneficiaries in December 2025,^11^ although the underlying population frame differs. From 2016 to 2025, Maryland’s HHS empower count for claims for electricity-dependent durable medical equipment increased from 28,939 to 35,575 when compared consistently in August of each year (+22.9%).^11^ These individuals face acute health risks during power disruptions and are not consistently integrated into emergency preparedness systems, increasing their vulnerability to both physical and psychological distress. Studies of Winter Storm Uri’s mental health impacts found high rates of psychological distress. 18% of 790 surveyed Texas residents reported post-traumatic stress; those experiencing both power and water outages combined with high COVID-19 impacts had 7.7 times higher odds of PTS (post-traumatic stress).^12^

Maryland provides a particularly relevant setting for this study because of its aging electricity infrastructure and increasing climate stress: Maryland’s electric grid has been described as “battered and getting worse” by state officials,^13^ with outages increasing due to aging infrastructure and more frequent severe weather events.^4^ In recent years, Maryland has retired 6,000 MW of power generation capacity while adding only 1,600 MW.^14^ PJM (Pennsylvania-New Jersey-Maryland) Interconnection has noted that “no major high-voltage lines constructed in recent memory” in Maryland as of 2024, leaving its transmission infrastructure increasingly strained.^15^ Consistent with this trend, the frequency of outage episodes lasting at least 1 h in our data increased at an average annual rate of approximately 10% over the study period (2018–2023). The state encompasses both dense metropolitan corridors in the Baltimore-Washington region and rural and coastal communities on the Eastern Shore and in Western Maryland, creating substantial geographic heterogeneity in infrastructure exposure and healthcare access: approximately two-thirds of the ZIP codes in our sample (321 of 490) fall within large metropolitan areas (population ≥1 million), while the remaining third encompasses smaller metro areas and rural communities. In addition, this study uses high-resolution ZIP code-level outage data, whereas much of the prior literature relies on county-level datasets,^16^^,^^17^^,^^18^ allowing more precise estimation of localized exposure and community-level heterogeneity. When linked to Healthcare Cost and Utilization Project (HCUP) State Inpatient Databases (SID) administrative records, these data make Maryland an empirical setting for identifying the effects of power outages on clinically diagnosed mental health outcomes.

Prolonged power outages not only threaten public health in socioeconomically disadvantaged communities but also involve longer restoration delays during extreme weather events for vulnerable populations.^19^ In the US., the severe outages in California, Texas, and Louisiana in 2020 and 2021 caused by extreme weather were estimated to cost $2 billion, $80–130 billion, and $31–44 billion, respectively.^20^ Recent evidence suggests that power outage burdens are unevenly distributed across communities.^21^ found that ZIP codes with lower median household income experienced more frequent outages—a disparity the authors attributed to spatial differences in grid infrastructure vulnerability—while outage durations were shorter in areas with critical facilities such as hospitals and fire stations, consistent with utilities’ practice of prioritizing restoration in those areas.^22^ found that areas with a high share of minority populations were more than four times more likely to suffer a blackout, and that low-income communities experience more frequent outages and less reliable electricity service. These disparities may be further compounded by aging electrical grid infrastructure; today, over 70% of the US electrical grid is over 25 years old, increasing the system’s vulnerability to extreme weather events and other outage causes.^23^

The prevalence of mental health disorders in the US has been surging over the last few years, affecting more than 22% of the adult population.^24^ Maryland faces a particularly acute mental health burden: the state’s mental health service utilization rate reached 42.2 per 1,000 population in 2023—nearly twice the national average of 24.5 per 1,000—with over 260,000 individuals receiving state mental health services, 78% of whom met the federal definition for serious mental illness or serious emotional disturbance.^25^ Maryland’s Medicaid-funded share of mental health clients (97%) also substantially exceeds the national average (76%), reflecting both higher need and greater reliance on public insurance for behavioral health care. In the US, major depressive disorder (MDD) affected 19.8 million adults in 2019, with an economic burden of $333.7 billion.^26^ Power outages are associated with psychological stress, anxiety, and disruption of daily life and communication.^27^^,^^28^ Evidence from the 2021 Texas winter storm and power crisis also suggests that mental health effects can continue after the immediate event.^7^ More broadly, recent evidence from Germany shows that energy poverty is strongly associated with deteriorations in mental rather than physical health, suggesting that inadequate access to reliable energy services can directly harm psychological well-being.^29^ As distress worsens, some patients could shift to emergency or inpatient care. Because mental and substance use-related hospital care is resource-intensive, greater hospital utilization can increase healthcare system burden and spending.^30^ This pattern is consistent with broader evidence that serious mental illness carries substantial long-term lifetime economic costs.^31^

Despite growing evidence, important gaps remain. Many studies rely on self-reported, short-term distress rather than clinically diagnosed outcomes.^32^ conducted a scoping review of 56 empirical studies on climate change and mental health; 23 (41% of studies) relied on self-reported survey data that focused on self-reported distress shortly after immediate extreme weather events, which may not capture clinically diagnosed or longer-term mental health impacts. In addition, most existing work provides limited causal identification of the independent effect of power outages, because outages frequently co-occur with extreme weather events that affect mental health through separate pathways.

This study provides the first empirical evidence of power outages on mental health using high-resolution power outage data and de-identified discharge-level clinical data—the administrative inpatient claims in Maryland and addresses these two gaps. First, we leverage de-identified administrative inpatient discharge records from the HCUP State Inpatient Database (2018–2023), linked with high-resolution power outage data, to measure clinically diagnosed mental health hospitalizations rather than self-reported distress. Using multi-year administrative hospitalization data, we find that more frequent outage episodes of moderate duration (1–3 h), rather than prolonged events, are associated with approximately a 1–2% increase in the rate of hospitalizations with a primary mental health diagnosis, measured at the ZIP code level. Second, to address endogeneity, we implement an instrumental variable strategy based on pre-existing electricity grid infrastructure—specifically, transformer density (distribution layer), substation density, and high-voltage transmission line density (transmission layer)—which exploits variation in the physical structure of the grid to identify a causal effect of power outages on mental health hospitalization rates. In addition, we integrate infrastructure stressors with social vulnerability factors and test their interaction effects on mental health risk, using ZIP code and time fixed effects, with results that are robust across specifications and stronger in more vulnerable communities.

Our findings contribute to the literature by shifting attention from rare, large-scale blackout events to more frequent, short-duration outages that characterize everyday grid operations. While often treated as routine operational issues, these interruptions are shown to be associated with measurable changes in mental health outcomes. At the same time, the heterogeneity patterns indicate that observed hospitalization effects are shaped by both underlying need and access to care. This implies that outage-related mental health burdens may be systematically under-recorded in communities with more limited healthcare access, highlighting a potential gap between observed outcomes and true population impacts.

### Our contribution

Extreme weather events and power outages frequently co-occur across the US regions,^33^ making it difficult to attribute mental health impacts to outages alone. Although both are linked to adverse mental health outcomes, they likely operate through distinct pathways: extreme weather, particularly high temperatures, can affect mental health through physiological channels such as thermoregulatory stress and sleep disruption,^34^^,^^35^^,^^36^ whereas power outages may affect mental health through infrastructure-dependent disruptions, including loss of thermal comfort, disrupted routines, and reduced access to social support,^28^^,^^37^ as well as interruptions in telehealth, medication access, and crisis support.^7^^,^^38^ However, prior empirical studies (Note S1) have largely focused on disaster contexts in which weather extremes and outages occur simultaneously, limiting causal identification of the independent effect of outages.^12^^,^^39^^,^^40^^,^^41^

Our study makes the following contributions. Most of the prior literature (See Note S1 for a detailed literature review) has largely examined the combined effects of co-occurring hazards rather than isolating the role of power outages, and has often relied on short-term event-based data that do not distinguish emergency department discharges from inpatient psychiatric admissions. This study addresses these limitations in two ways. First, unlike most prior studies that mainly relied on survey-based data to measure mental health^32^ rather than using clinical and administrative data, as we do here, our study leverages comprehensive administrative data from the Maryland HCUP SID (State Inpatient Database), allowing us to quantify the mental health burden of power outages more precisely by tracking post-ED hospitalization outcomes and to control for a richer set of temporal and geographic covariates. Administrative hospital data offer several advantages: they capture objectively diagnosed conditions using standardized International Classification of Diseases, 10^th^ Revision (ICD-10-CM, Method S1) coding, and provide population-level coverage of all inpatient admissions within the state. Self-reported mental health measures, such as those collected from surveys, are prone to reporting bias and may not accurately represent the severity of mental health disorders, as they are influenced by respondents’ perceptions and their willingness to disclose mental health issues. These surveys are inadequate for capturing the time impact of power outages on a large scale. To address these limitations, our analysis focuses on patients whose primary diagnosis is a mental health condition, including all diagnoses listed in (Method S1). The primary diagnosis reflects the main reason for admission or treatment, which allows us to isolate acute mental health cases directly attributable to the underlying condition (cases where mental health is the main and underlying condition of the patient) rather than secondary or comorbid pathways. This approach thus provides a cleaner attribution of mental health outcomes to power outage events, minimizing confounding from unrelated physical health factors.

Second, estimating the causal impact of power outages on mental health poses significant challenges, primarily due to confounding factors—both observed and unobserved—that affect both exposure and outcomes. Using a large and statewide hospital admission database that covers the entire state of Maryland, this study (1) evaluates the causal impact of power outages on mental health hospitalizations as primary diagnosis; (2) investigates whether the relationship between outages and mental health varies across sociodemographic groups. To identify the causal effect of power outages on mental health admissions, we employ an identification strategy that accounts for multiple potential confounding pathways. Specifically, we adjust for time-varying weather conditions and demographic factors, include ZIP-code and time fixed effects, and implement an instrumental-variable (IV) approach based on electricity-grid infrastructure (e.g., transformer density and distribution-line density; with alternative specifications using substation density and high-voltage transmission-line density) to address potential endogeneity in outage exposure.

## Results

### Baseline estimates: Power outages and mental health hospitalizations

**Figure 1:**
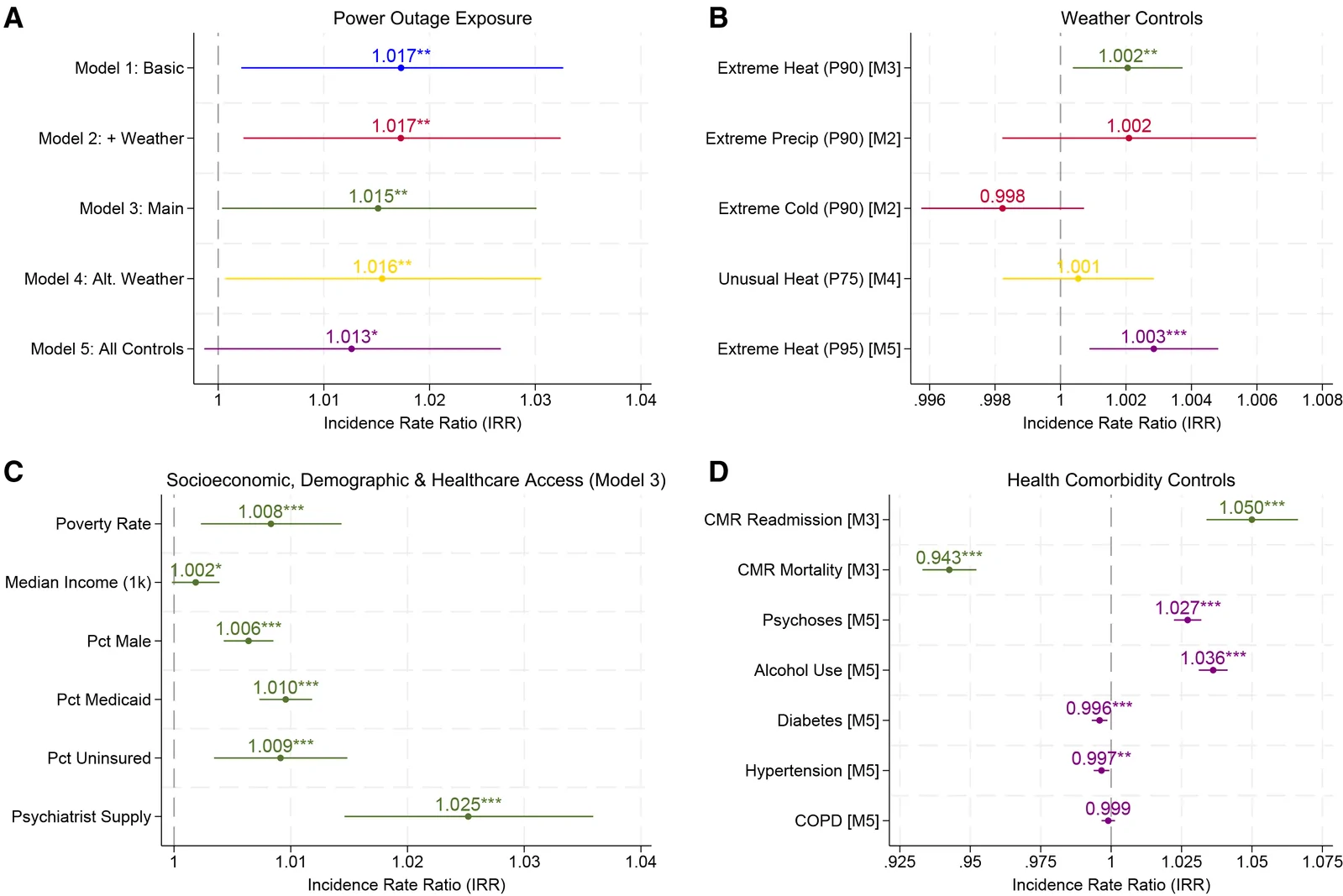
Baseline PPML coefficient estimates—mental health hospitalizations and power outages Note: Each image presents incidence rate ratios (IRRs) with 95% confidence intervals from PPML-HDFE regressions. (A) shows the main effect of power outage exposure (ln_outage_1hr_p75) across five model specifications. (B) shows weather controls from the respective model specifications. (C) shows socioeconomic, demographic, and healthcare access controls from Model 3 (M3). (D) shows health comorbidity controls (Elixhauser comorbidity indices from Model 3; disease prevalence from Model 5). Model 1 (M1) includes poverty rate, median household income, and case-mix-adjusted comorbidity and mortality indices (CMR). Model (M2) adds extreme heat, extreme precipitation, and extreme cold days (all at the 90^th^ percentile threshold). Model 3 (M3) retains extreme heat (90^th^ percentile) and adds demographic controls (percent male, percent Black, percent Asian), insurance coverage (Medicaid share, uninsured share), and psychiatrist supply. Model 4 (M4) is identical to M3 but replaces the 90th percentile heat threshold with the 75th percentile. Model 5 (M5) uses the 95th percentile heat threshold, replaces the CMR comorbidity index with disease-specific prevalence indicators (psychoses, alcohol use disorders, diabetes, hypertension, COPD), and retains the CMR mortality index. All models include ZIP code and Year×Quarter fixed effects with total population as exposure. Standard errors clustered at the ZIP code level. ∗*p* < 0.10, ∗∗*p* < 0.05, and ∗∗∗*p* < 0.01. See Table S9 for full regression results.

Figure 1 presents regression results from Poisson pseudo-maximum likelihood models with high-dimensional fixed effects (PPML-HDFE), which regress ZIP-quarter-level mental health hospitalization rates on log-transformed power outage exposure—measured as the number of outage episodes per ZIP-quarter that lasted at least 1 h and affected more than the 75^th^ percentile of customers (see power outage data in the STAR Methods section and Method S2 for variable definition and construction details)—across five progressively richer specifications that sequentially add weather, socioeconomic, demographic, and health-system covariates. All models include ZIP code and year-by-quarter fixed effects to account for time-invariant local characteristics and absorb shocks common to all ZIP codes in a given year-quarter. The identification strategy relies on within-ZIP variation over time in power outage exposure, under the assumption that—conditional on fixed effects and observed covariates-the timing of outages is exogenous to local mental health needs, reporting behavior, or hospital utilization. A positive, stable association across all specifications is the main finding of the baseline analysis.

Figure 1 presents the PPML-HDFE regression results across five specifications, with all models including ZIP code and year-by-quarter fixed effects and using total population as the exposure term. The positive association between power outage exposure and mental health hospitalizations is robust across all specifications (A). The estimated IRR for ln_outage_1hr_p75 (the natural log of one plus the count of outage episodes lasting at least 1 hr and exceeding the 75^th^ percentile of outage intensity; see power outage data in the STAR Methods section and Method S2 for variable definition and construction details) ranges from 1.013 to 1.017, indicating that a one-unit increase in log outage exposure—approximately a 2.7-fold increase in severe outage episodes (e.g., from 5 to 14 per quarter)—is associated with a 1.3–1.7% increase in mental-health-related hospitalization rates. Beginning with a basic specification including socioeconomic controls and comorbidity indices (Model 1, IRR = 1.017, *p* < 0.05), the effect remains stable as we add weather controls (Model 2, IRR = 1.017, *p* < 0.05), demographic and healthcare access variables (Model 3, IRR = 1.015, *p* < 0.05), an alternative heat threshold at the 75^th^ percentile (Model 4, IRR = 1.016, *p* < 0.05), and a set of disease-specific comorbidity indicators (Model 5, IRR = 1.013, *p* < 0.10). This stability indicates that the estimated effect is robust to the inclusion of observable confounders.

Among the environmental controls, extreme heat emerges as a significant risk factor for mental health admissions (B). Extreme heat days at the 90th percentile show a significant positive association with primary mental health hospitalization rates (IRR = 1.003, *p* < 0.01 in Model 2; IRR = 1.002, *p* < 0.05 in Model 3), and the effect remains significant when using the more stringent 95th percentile threshold in Model 5 (IRR = 1.003, *p* < 0.01). In contrast, extreme precipitation and extreme cold days show no statistically significant associations in Model 2, and the lower heat threshold at the 75th percentile is also not significant in Model 4, suggesting that only more extreme temperatures meaningfully affect mental health outcomes. Table S15 further confirms this pattern by re-estimating each specification under all three heat thresholds; the outage coefficient is essentially unchanged regardless of the threshold used. Importantly, the power outage effect remains significant even after controlling for these weather variables, confirming that outages exert an independent impact on mental health beyond weather-driven stress. Beyond the main outage effect, the control variables help characterize factors shaping mental health hospitalization patterns across Maryland ZIP codes (Figure 1, 1C and 1D; see Table S9 for full results). A clear pattern emerges around healthcare access. Areas with more psychiatric providers per capita are associated with higher—not lower—mental health admission rates (IRR = 1.025–1.028, *p* < 0.01), consistent with greater case identification and referral rather than higher underlying burden.

Similarly, ZIP codes with higher Medicaid enrollment (IRR = 1.006–1.010, *p* < 0.01) and uninsured rates (IRR = 1.009, *p* < 0.01 in main models) show significantly higher rates of primary mental health hospitalizations. This pattern is consistent with the idea that populations with more limited access to outpatient or preventive care may rely more heavily on crisis-driven inpatient services. Socioeconomic disadvantage is consistently associated with higher hospitalization rates. For every 1% point increase in the poverty rate, mental health admissions increase by 0.6–0.8% (IRR = 1.006–1.008), while median household income shows a small positive association in Models 3 and 4 (IRR = 1.002, *p* < 0.10)—a pattern that may reflect better access to care and diagnosis in higher-income areas rather than a higher underlying burden of mental illness. Demographic composition also matters: ZIP codes with a higher share of male residents show consistently higher primary mental health admission rates (IRR = 1.005–1.006, *p* < 0.01). Areas with larger Asian populations have substantially lower primary mental health admission rates (IRR = 0.331–0.340, *p* < 0.05), even after controlling for other factors. The percentage of Black residents is not statistically significant after controlling for other variables (IRR = 0.970–0.983).

The health comorbidity controls capture an important dimension of population vulnerability. Areas with higher average Elixhauser comorbidity readmission risk—capturing a greater burden of underlying comorbidities (see Method S5 for details)—exhibit significantly higher mental health hospitalization rates (IRR = 1.049–1.050, *p* < 0.01 in Models 1–4). This pattern suggests that populations with more complex underlying health conditions may be more vulnerable to mental health-related hospitalizations. Conversely, areas with higher mortality risk exhibit lower mental health hospitalization rates (IRR = 0.939–0.972, *p* < 0.01). When disease-specific indicators are included in Model 5, areas with higher prevalence of psychoses (IRR = 1.027, *p* < 0.01) and alcohol-related disorders (IRR = 1.036, *p* < 0.01) show significantly higher admission rates, whereas physical conditions such as diabetes with complications (IRR = 0.996, *p* < 0.01) and hypertension with complications (IRR = 0.997, *p* < 0.05) are negatively associated. COPD (Chronic Obstructive Pulmonary Disease) shows no significant association (IRR = 0.999).

### Alternative definitions of outage exposure: Robustness and sensitivity

**Figure 2:**
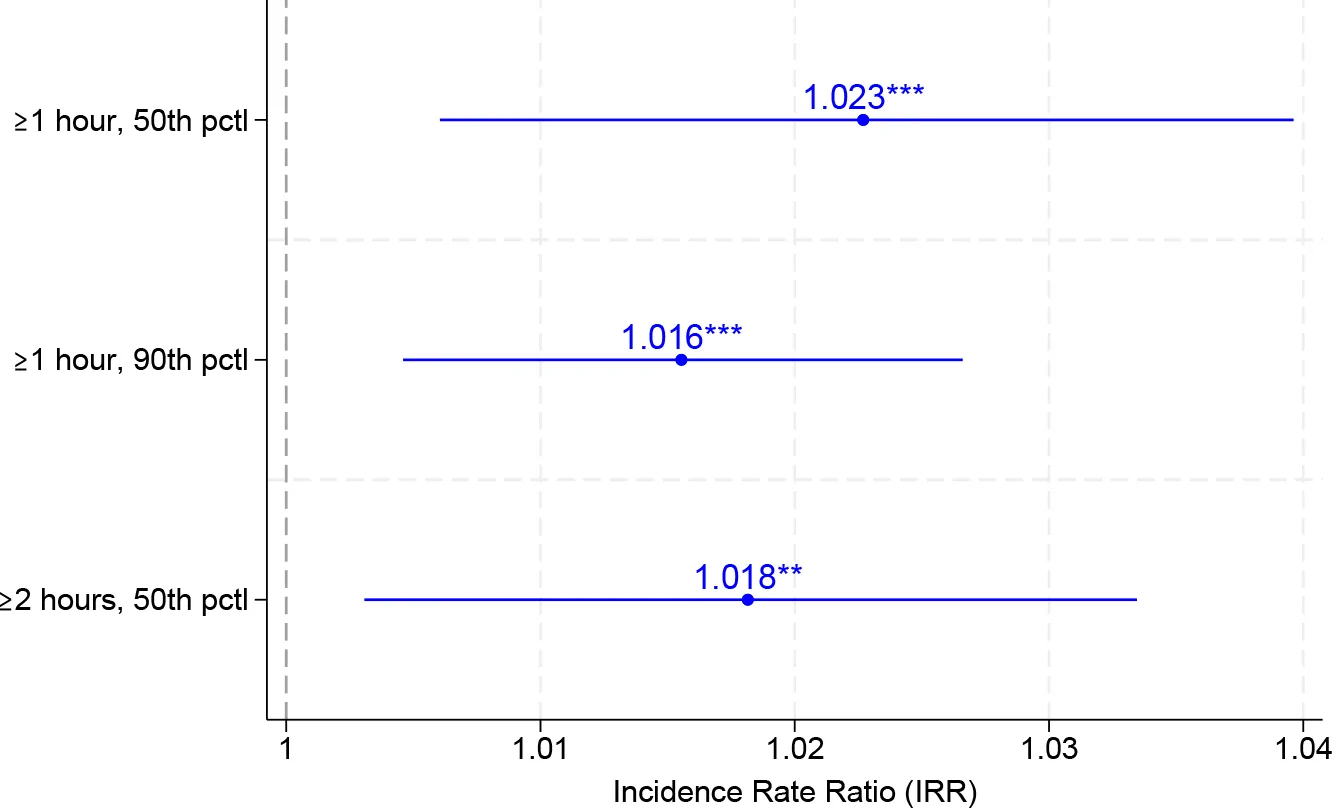
Robustness – power outage effect across alternative threshold definitions Note: Each point shows the incidence rate ratio (IRR) from a separate PPML regression using alternative outage threshold definitions. Horizontal bars represent 95% confidence intervals. ∗∗*p* < 0.05 and ∗∗∗*p* < 0.01. All models include ZIP code and Year x Quarter fixed effects with the full set of controls. See Table S10 for full results.

We assess whether the estimated outage-mental health relationship is sensitive to alternative definitions of outage exposure. In the baseline specification, outage exposure is defined as episodes lasting at least 1 hr and exceeding the 75th percentile of severity, consistent with percentile-based definitions used in prior studies (Table 1). To evaluate sensitivity, Figure 2 checks the robustness under three alternative threshold definitions that vary both the minimum duration and severity cutoff. All specifications retain the full set of controls, ZIP code, and Year × Quarter fixed effects, and population exposure.

**Table 1:** Summary of power outage definitions

Reference	Power outage definition	Location	Time period	Resolution
Zhang et al.^42^	% customers affected, and Binary variable: yes/no %customers affected >50^th^ percentile	New York State	2001–2013	Daily
Lin et al.^40^	binary variable: yes/no %customers affected >50^th^ percentile	New York State	2001–2013	Daily
Sheridan et al.^41^	binary variable: yes/no % customers affected >50^th^ percentile	New York State	2005–2013	Daily
Xiao et al.^43^	binary variable: yes/no % customers affected >50th percentile	New York State	Oct 29, 2012 to Nov 27, 2012	Daily
Deng et al.^44^	binary variable: yes/no % customers affected >75^th^ percentile	New York State	2002–2018	Daily
Do et al.^33^	% customers affected	Nation-wide, county level	2018–2020	Hourly
Flores et al.^16^	% customers affected	New York State	2017–2020	Hourly

The results (Figure 2) are consistent across specifications. Defining outages as events lasting at least 1 h above the 50th percentile of severity yields an IRR of 1.023 (*p* < 0.01), implying a 2.3% increase in mental health hospitalization rates per unit increase in log outage exposure. Increasing the severity threshold to the 90th percentile produces a slightly smaller but more precisely estimated effect (IRR = 1.016, *p* < 0.01). Extending the minimum duration to 2 h while maintaining the 50th percentile cutoff yields a nearly identical estimate (IRR = 1.018, *p* < 0.05). Across all definitions, point estimates fall within a narrow 1.6–2.3% range, indicating that the estimated relationship is not driven by any single threshold choice. Full regression results are reported in Table S10.

**Figure 3:**
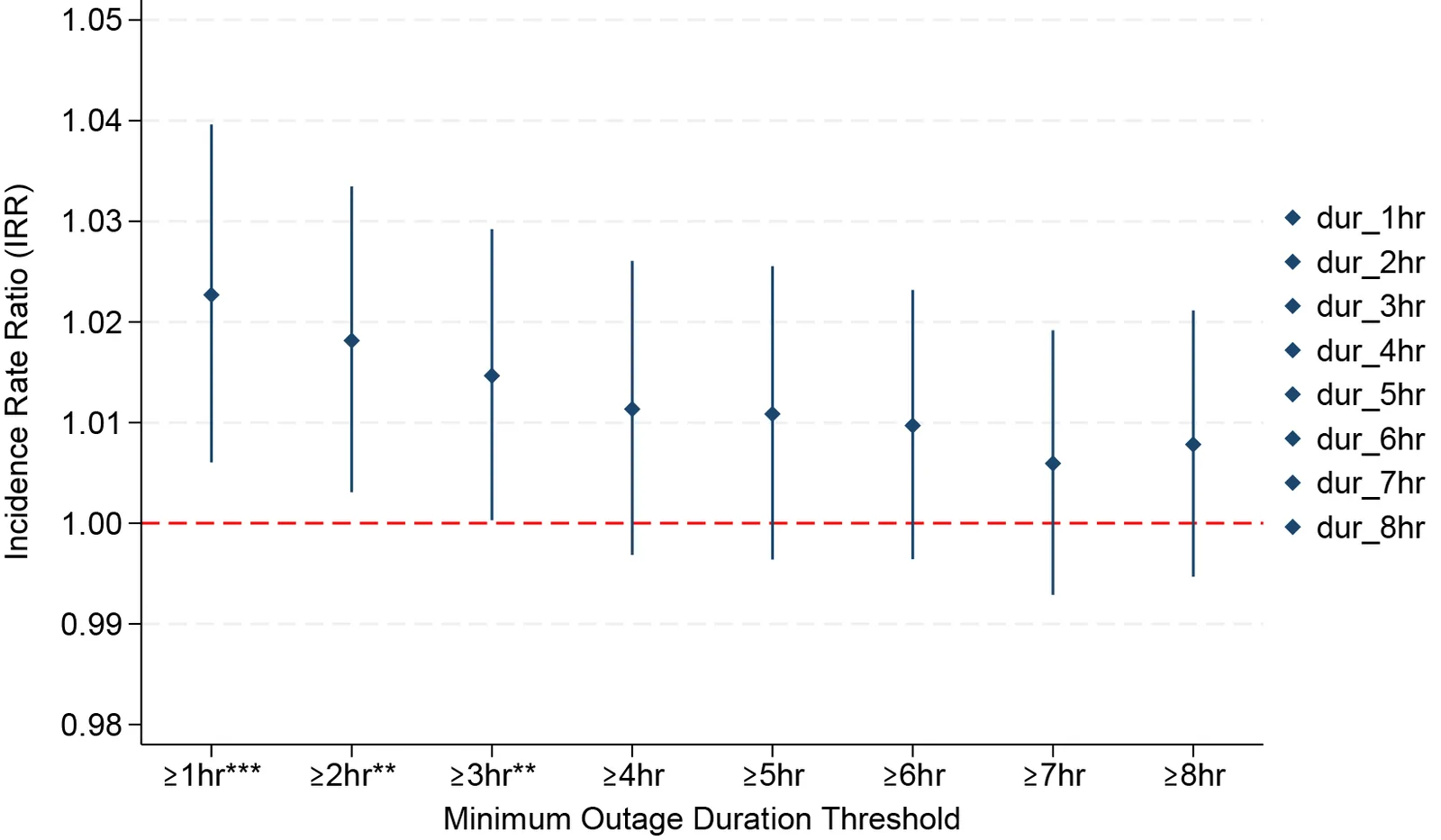
Sensitivity test Note: Each point shows the incidence rate ratio (IRR) from a separate PPML regression. The outage measure is defined as ln(1 + episodes), where episodes are outage events lasting ≥ X hours and above the 50th percentile of severity within each ZIP-quarter. 95% confidence intervals are shown. ∗*p* < 0.10, ∗∗p < 0.05, ∗∗∗p < 0.01. All models include ZIP and Year × Quarter fixed effects. Summary of the statistics for outage episodes by duration threshold (Log-transformed) can be found in Table S11.

We further examine sensitivity to the minimum outage duration threshold by estimating separate PPML regressions with outage exposure defined as ln(1 + episodes), where episodes correspond to outages lasting at least X hours and exceeding the 50th percentile of severity within each ZIP-quarter (Figure 3) (see power outage data in the STAR Methods section and Method S2 for details on exposure variable construction). The estimated effects are largest for shorter-duration outages. Outages lasting at least 1 h yield an IRR of approximately 1.023 (*p* < 0.01). The estimated effect declines gradually as the duration threshold increases, with IRRs of about 1.018 (*p* < 0.05) at 2 h and 1.015 (*p* < 0.05) at 3 h. For thresholds of 4 h and above, the estimates are smaller in magnitude and no longer statistically distinguishable from zero, with confidence intervals overlapping the null value of 1. Shorter outages are substantially more frequent than longer-duration events (Table S11), suggesting that the loss of statistical significance at higher thresholds likely reflects reduced statistical power rather than a reversal of the underlying relationship.

Taken together, these robustness and sensitivity analyses indicate that the positive association between power outages and mental health hospitalizations is not driven by any single threshold definition. The results are consistent across alternative severity cutoffs and duration thresholds, with effects concentrated among relatively frequent, moderate-duration outages (1–3 h), rather than rare, prolonged events (Figure 3). This pattern suggests that even short-duration outages can accumulate over time and contribute meaningfully to mental health burdens in affected communities. Overall, the findings highlight the importance of outage frequency, in addition to event severity, in understanding the mental health impacts of power infrastructure disruptions.

As an additional specification check, replacing ZIP-code fixed effects with city-by-year fixed effects—which absorb city-level time-varying unobservables such as local policy and economic shifts—yields a consistent and somewhat larger estimate (IRR = 1.031, 95% CI: 1.007–1.056, *p* = 0.012), confirming that the positive association is not driven by city-level confounders that evolve over the study period (see Table S12 for full result).

### Effect modification by extreme heat

To examine whether the association between power outages and mental health hospitalizations is amplified during periods of extreme heat, we estimate an augmented PPML-HDFE model that incorporates an interaction between log outage exposure and extreme heat days, both mean-centered at the sample mean. This specification tests whether concurrent thermal stress and electricity disruption compound the psychological burden beyond the sum of their individual effects.

The interaction term (Table 2) is not statistically significant (IRR = 1.000, 95% CI: 1.000–1.001, *p* = 0.434; joint test: *χ*^2^(1) = 0.61, *p* = 0.434), suggesting that the mental health impact of power outages does not significantly differ across levels of extreme heat exposure. The main effect of outage exposure remains positive (IRR = 1.015, *p* = 0.050), consistent with the baseline specification, and extreme heat days are independently associated with higher mental health hospitalization rates (IRR = 1.002, *p* = 0.025).

**Table 2:** Effect modification by extreme heat: outage × heat interaction (PPML-HDFE, ZIP-quarter level)

Variable	IRR	95% CI	*p* value
Log outage hours (centered)	1.015	1.000–1.030	0.050
Extreme heat days (centered)	1.002	1.000–1.005	0.025
Outage × heat (interaction)	1.000	1.000–1.001	0.434
N (observations)	8,152	–	–
Clusters (ZIP codes)	430	–	–

Estimates from PPML-HDFE model with ZIP code and year × quarter fixed effects and population offset. Log outage hours and extreme heat days are both mean-centered. IRR = incidence rate ratio. Joint test of interaction term: χ^2^(1) = 0.61, *p* = 0.4338. Full covariate set matches baseline model. Standard errors clustered at ZIP-code level. See also Table S15 for the outage effect estimated under alternative extreme-heat thresholds.

### Instrumental variable results

**Figure 4:**
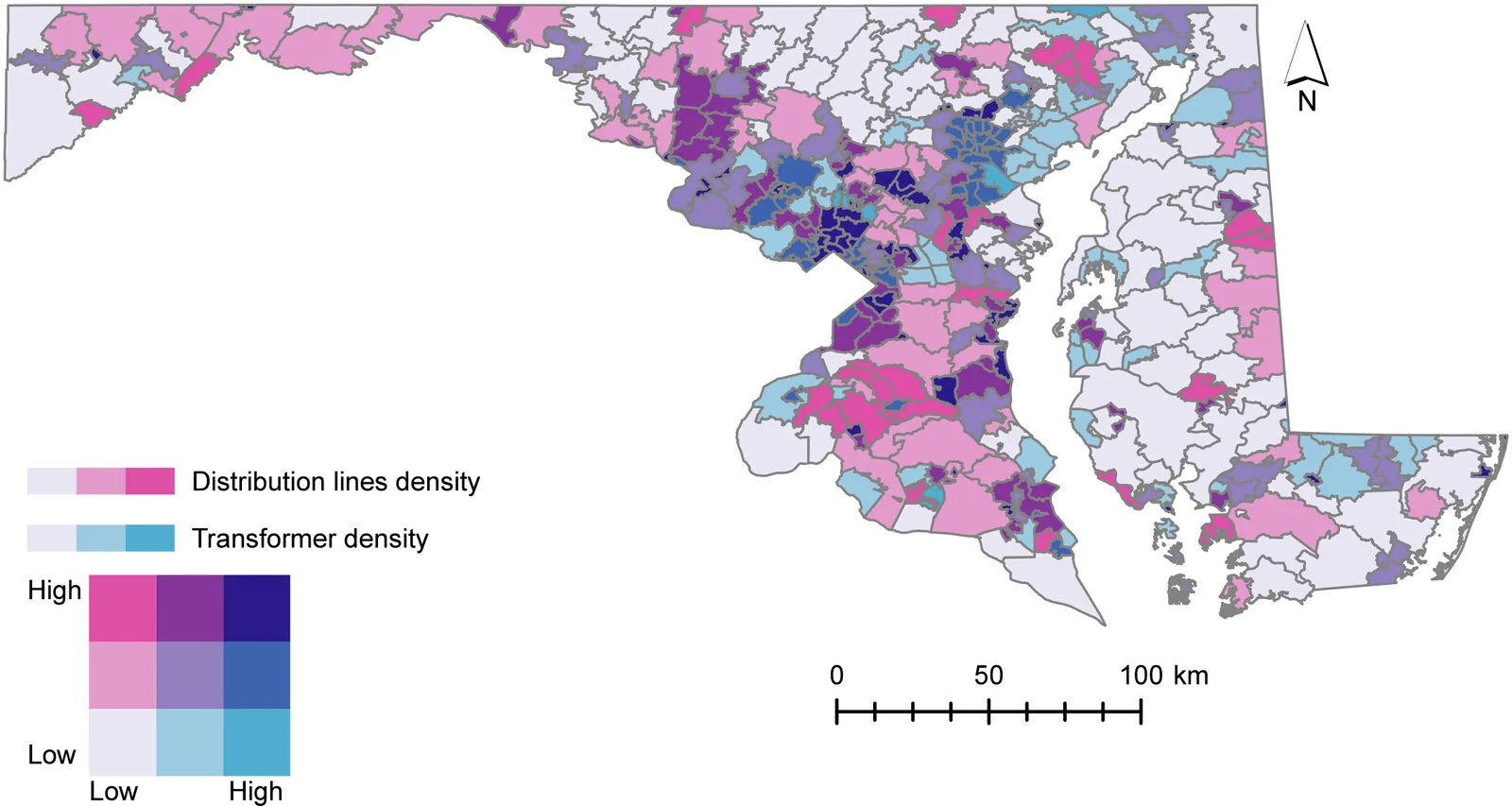
Spatial distribution of power infrastructure density across Maryland ZIP codes This figure presents a bivariate classification of distribution line density (pink) and transformer density (blue) at the ZIP code level. Combined colors (purple tones) indicate areas where both densities are high. Color intensity reflects relative density levels based on quantile classification, highlighting substantial geographic variation in local electricity infrastructure capacity across Maryland. See Method S6 for details on the construction of the infrastructure density measures. Scale bar, 100 km.

**Figure 5:**
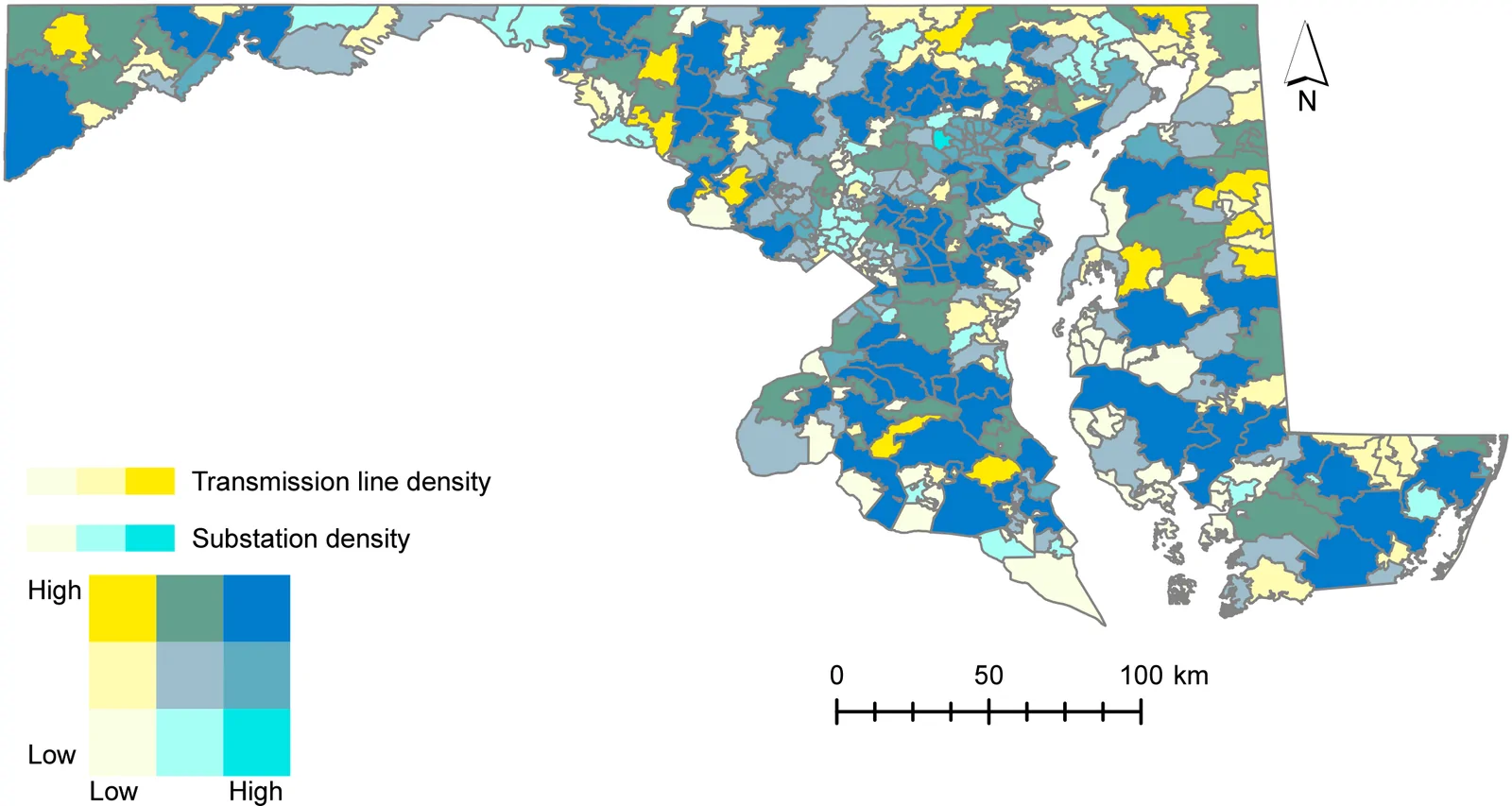
Spatial distribution of transmission line and substation density across Maryland ZIP codes Bivariate choropleth map displaying the joint distribution of transmission line density (yellow) and substation density (blue) across Maryland ZIP codes. Dark blue-green areas indicate high density of both infrastructure types; yellow areas have high transmission line but low substation density; blue areas have high substation but low transmission line density; pale/cream areas indicate low density of both. See Method S6 for details on the construction of the infrastructure density measures. Scale bar, 100 km.

To address potential endogeneity in outage exposure, we implement an instrumental variable (IV) approach using three measures of electricity grid infrastructure—transformer density, substation density, and high-voltage transmission line density (see Figures 4 and 5 for the spatial distribution of these infrastructures across Maryland ZIP codes, and Method S6 for details on construction of the instrumental variables)—as instruments for power outage exposure. Our IV estimates confirm a positive and statistically significant effect of power outage exposure on mental health hospitalizations, consistent in direction with the baseline PPML results, lending support to a causal interpretation that power outages adversely affect mental health outcomes (see STAR Methods for detailed instrument construction, identification assumptions, and the physical architecture of the power grid).

The first-stage regressions confirm (Table 3) that grid infrastructure predicts outage exposure. Both transmission-level and distribution-level infrastructure densities are significantly associated with the frequency of severe outage episodes. This supports the relevance condition: areas with grid structures that are more susceptible to cascading or equipment-level failures indeed experience more outage exposure.

**Table 3:** IV Poisson estimates — summary of main results

	(1)	(2)	(3)
	Transformer IVs	Substation and transmission lines as IVs	Transformer, substation, and transmission lines as IVs
**Panel A:Secondstage**			

ln_outage_1hr_p75	0.420∗∗∗ (0.142)	0.301∗ (0.166)	0.380∗∗∗ (0.120)

**Panel B:Firststage**			

dens_transformer	−0.116∗∗∗ (0.037)	–	−0.102∗∗∗ (0.029)
dens_substation	–	1.706∗∗∗ (0.601)	1.640∗∗∗ (0.595)
dens_transmission_line	–	0.189∗∗∗ (0.067)	0.177∗∗∗ (0.066)
Observations	8,204	8,204	8,204
First-stage F	9.80	21.32	19.76
First-stage *p* value	<0.001	<0.001	<0.001
Controls	yes	yes	yes
Year FE	yes	yes	yes
Hansen J *p* value	–	0.383	0.556

The table reports second-stage IV Poisson GMM estimates and first-stage coefficients for excluded instruments. Column (1) uses transformer density as the sole excluded instrument. Column (2) uses substation density and transmission line density as IVs. Column (3) uses all three infrastructure variables—transformer density, substation density, and transmission line density—as IVs. All models include year fixed effects and controls for extreme heat days, socioeconomic characteristics, insurance coverage, psychiatrist supply, comorbidity indices, and racial composition. Standard errors are clustered at the ZIP code level. ∗*p* < 0.10, ∗∗*p* < 0.05, ∗∗∗*p* < 0.01. It should be noted that Column (1), using transformer density as the sole instrument, yields a first-stage F-statistic of 9.80—marginally below the conventional weak-instrument threshold^45^ of approximately 10—indicating borderline instrument strength. See also Figure S1 and Note S2.

Table 3 presents IV Poisson estimates using three alternative instrument sets. In Column (1), transformer density alone serves as the excluded instrument and is negatively associated with outage intensity (−0.116, *p* < 0.01), consistent with denser local networks being better able to localize faults; the F-statistic of 9.80 indicates adequate instrument strength. Column (2) uses upstream transmission infrastructure—substation density and transmission line density—both positively associated with outage exposure (1.706 and 0.189, both *p* < 0.01), reflecting the greater cascade failure risk in highly interconnected bulk power networks; the F-statistic of 21.32 confirms strong instruments. Column (3) combines all three variables: transformer density remains negatively associated (−0.102, *p* < 0.01), while substation density and transmission line density remain positive (1.640 and 0.177, both *p* < 0.01), with a joint F-statistic of 19.76.

In the second stage, all three specifications yield positive and statistically significant effects of outage exposure on mental health hospitalizations, with consistent direction across instrument sets. The coefficient on ln_outage_1hr_p75 is 0.420 (*p* < 0.01) using the transformer IV (Column 1), 0.301 (*p* < 0.10) using transmission IVs (Column 2), and 0.380 (*p* < 0.01) using all three IVs (Column 3). Because the model is estimated in exponential mean form, these coefficients imply that a one-unit increase in log outage exposure is associated with approximately a 35–52% increase in mental-health-related hospitalizations (IRR ≈1.35–1.52). The Hansen J test for overidentification does not reject instrument validity in either of the overidentified specifications (*p* = 0.383 for Column 2; *p* = 0.556 for Column 3), strongly supporting the exogeneity of the instruments. The consistency of the estimates across downstream and upstream infrastructure measures—which capture distinct outage mechanisms—provides robust evidence that power outages increase mental health hospitalizations.

While the IV estimates are substantially larger than the PPML estimates, the implied effect size remains plausible when translated into absolute terms. The IV coefficient of 0.380 (IRR = 1.46) implies that a one-unit increase in log outage exposure—equivalent to approximately tripling the number of outage episodes (e.g., from 3 to 8 episodes per quarter)—is associated with a 46% increase in mental health hospitalizations. Given the mean baseline rate of 16.4 primary mental health hospitalizations per ZIP-quarter, this translates to approximately 7–8 additional cases (16.4 × 0.46 ≈ 7.5). However, the distribution of hospitalizations is highly right-skewed, with a median of only 4 cases per ZIP-quarter. For a typical ZIP code at the median, a 46% increase would imply approximately 1.8 additional hospitalizations. Moreover, the IV estimates (IRR ranging from 1.35 to 1.52 across instrument sets) are substantially larger than the baseline PPML estimates (IRR ≈1.013–1.017). A cross-sectional Poisson specification without ZIP fixed effects but with the full set of controls yields an IRR of only 1.037, suggesting that the absence of ZIP fixed effects contributes minimally to the magnitude difference. We therefore interpret the PPML estimates as a conservative lower bound, and the IV estimates as an upper bound on the true effect; both approaches consistently confirm a positive and statistically significant effect of power outage exposure on mental health hospitalizations. The IV results are robust to alternative outage definitions. Across seven specifications varying episode duration (1–8 h) and severity thresholds (P50 and P75), all IV estimates remain positive, with five reaching statistical significance (IRR = 1.46–2.01), particularly at shorter duration thresholds. All Hansen J tests pass (*p* > 0.40; see Note S2 and Figure S1 for details).

### Healthcare access, socioeconomic status, racial composition, and seasonal heterogeneity

To assess whether the relationship between power outages and mental health hospitalizations varies across communities, we examine heterogeneity along five dimensions: urban-rural classification, poverty level, Medicaid coverage, seasonality, and gender composition. Urban-rural status is classified using the USDA Rural-Urban Continuum Codes (RUCC): large metropolitan areas (RUCC = 1, counties in metropolitan areas with a population of 1 million or more) versus all other areas. High-poverty ZIP codes are defined using the US Census Bureau threshold of a poverty rate ≥20%. High Medicaid coverage is defined as ZIP codes where at least one-quarter of hospitalized patients are covered by Medicaid, a threshold close to the national average of approximately 25%.^46^ Warm season includes April-September (quarters 2–3) and cold season includes October-March (quarters 1 and 4). Gender composition is defined using a median split of the ZIP code-level share of male patients.

**Figure 6:**
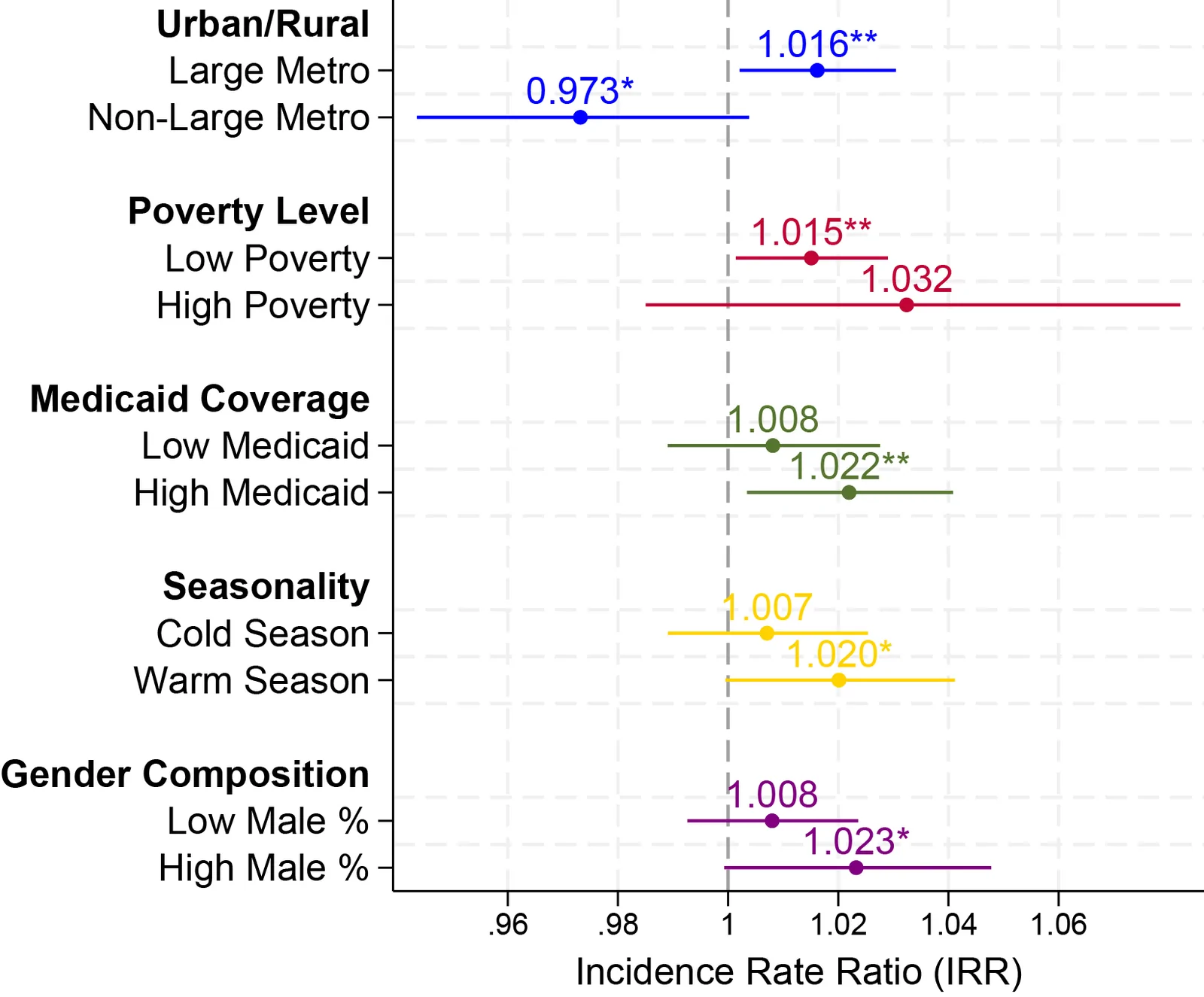
Heterogeneity analysis: power outage effects on mental health hospitalizations Each point represents the incidence rate ratio (IRR) for the outage variable (ln_outage_1hr_p75) from separate PPML-HDFE regressions estimated within subgroups, with 95% confidence intervals shown as horizontal bars. The dashed vertical line marks IRR = 1 (no effect). Urban-rural status is classified using USDA Rural-Urban Continuum Codes: Large Metro areas (RUCC = 1, counties in metropolitan areas with ≥1 million population) versus all other areas. High Poverty is defined using the US Census Bureau threshold of a poverty rate ≥20%. Medicaid coverage subgroups are defined by a median split of the ZIP code-level share of Medicaid-covered discharges, with High Medicaid defined as ≥25%. Warm Season includes Q2–Q3 (April-September) and Cold Season includes Q1 and Q4 (January-March and October-December) (see Table S13). All models include ZIP code and Year × Quarter fixed effects, and use total population as the exposure variable. Controls include extreme heat days, substance use prevalence, comorbidity burden, unemployment, racial composition, insurance coverage, and psychiatrist supply, with minor adjustments by dimension (e.g., extreme heat days are excluded from seasonal models to avoid collinearity; Medicaid and uninsured shares are replaced by median income in Medicaid coverage models to avoid mechanical correlation with the grouping variable). Standard errors are clustered at the ZIP code level. ∗*p* < 0.10 and ∗∗*p* < 0.05.

Based on urban-rural status, the estimated effects are concentrated in large metropolitan areas. In large metro area ZIP codes, outages are associated with a 1.6% increase in mental health hospitalizations (IRR = 1.016, *p* < 0.05) (Figure 6). In contrast, the estimate for non-large-metro areas is negative and marginally significant (IRR = 0.973, *p* < 0.10). Formal tests confirm that this is the only dimension along which the subgroup difference is statistically significant (*z* test *p* = 0.013; interaction *p* = 0.015) (see Table S13). One plausible explanation is that residents in large metropolitan areas have better access to hospital-based care, including emergency and psychiatric services, making them more likely to seek treatment following outage-related stress. By contrast, residents in smaller metro and rural areas may face greater barriers to inpatient care or rely more on informal support networks, which could lead to fewer recorded hospitalizations even if underlying distress increases.

A similar pattern emerges when stratifying by poverty level using the US Census Bureau threshold of 20%. The effect of outages is statistically significant in lower-poverty areas (IRR = 1.015, *p* < 0.05), while the point estimate in higher-poverty areas is larger but estimated with wider confidence intervals and not statistically significant (IRR = 1.032, *p* > 0.10), likely reflecting the relatively small number of ZIP codes exceeding the 20% poverty threshold in Maryland. The interaction model suggests a marginally significant difference between subgroups (*p* = 0.084), though the *z* test does not confirm this (*p* = 0.499). Although the effect reaches statistical significance only in lower-poverty areas, the point estimate is positive and numerically larger in higher-poverty areas, and the two subgroup estimates are not statistically distinguishable. We therefore interpret the difference in statistical significance as reflecting the small number of higher-poverty ZIP codes rather than an effect limited to lower-poverty communities.

When stratifying by Medicaid coverage, ZIP codes with higher Medicaid coverage (≥25% of patients covered by Medicaid) show a significant association between outages and primary mental health hospitalizations (IRR = 1.022, *p* < 0.05), whereas low-Medicaid areas show a smaller and non-significant effect (IRR = 1.008, *p* > 0.10). This pattern is consistent with greater vulnerability among communities with higher shares of publicly insured patients. However, formal tests indicate that this subgroup difference is not statistically significant (*z* test *p* = 0.313; interaction *p* = 0.483). We also examine whether the outage effect varies by season. The estimate is larger in the warm season (IRR = 1.020, *p* < 0.10) than in the cold season (IRR = 1.007, *p* > 0.10), consistent with a heat-related mechanism in which outages during warmer months disrupt access to air conditioning, increasing thermal stress that may exacerbate mental health conditions. However, the seasonal difference is not statistically significant (*z* test *p* = 0.353; interaction *p* = 0.223). Finally, when stratifying by gender composition (median split of the ZIP code-level share of male patients), the effect is somewhat larger in areas with a higher male share (IRR = 1.023, *p* < 0.10) than in areas with a lower male share (IRR = 1.008, *p* > 0.10), though the difference is not statistically significant (*z* test *p* = 0.297; interaction *p* = 0.907).

### Mental health as a secondary diagnosis

To examine whether the effects extend beyond admissions in which mental health is the principal diagnosis, we construct an additional outcome that counts admissions with a non-mental-health principal diagnosis but at least one mental health code (ICD-10 F00–F99) among the secondary diagnosis fields (DX2–DX101; see STAR Methods). These admissions are common, accounting for 38.4% of hospitalizations without a primary mental health diagnosis (see Table S2 for full counts).

To examine whether outage-related hospitalizations also appear when mental health is recorded as a secondary diagnosis rather than the principal reason for admission, we estimate additional PPML-HDFE models for two contrast outcomes: admissions with a non-mental-health principal diagnosis but at least one mental health code in a secondary diagnosis field, and admissions with no mental health code in any diagnosis position. Because these outcomes are constructed from secondary diagnosis fields, the baseline specification excludes the two Elixhauser comorbidity indices to avoid adjusting for controls derived from overlapping diagnosis-code information. Including them could mechanically absorb variation related to the secondary-diagnosis outcomes. Full construction details and complete results for this secondary-diagnosis analysis are reported in Note S4.

Power outages are not associated with mental-health-comorbid admissions in this specification (IRR = 0.996, 95% CI: 0.988–1.004; Table S16). This null result is unchanged when the Elixhauser indices are retained as a sensitivity check (IRR = 0.999). Admissions with no mental health code show only a small positive association with outages (IRR = 1.007, *p* < 0.05). The null result for mental-health-comorbid admissions is also unchanged across 24 alternative outage definitions: duration thresholds of 1–8 h combined with 50th, 75th, and 90th severity percentiles all yield IRRs close to 1.00 (0.996–1.001) and statistically non-significant.

These results suggest that, in our quarterly hospitalization data, the detectable short-run association with outages is concentrated in admissions where mental health is the principal diagnosis. Among vulnerable physical-health populations—diabetes, COPD/asthma, heart disease, dementia, and chronic kidney disease—point estimates are larger for admissions with coexisting depression or anxiety in three of the five groups (diabetes, dementia, and chronic kidney disease), but these subgroup estimates have wide confidence intervals because of smaller sample sizes (Table S17). Taken together, the secondary-diagnosis analyses do not rule out physical-health or comorbidity pathways. Rather, they suggest that such pathways may occur on shorter time scales than our quarterly data can resolve, whereas admissions with a principal mental health diagnosis remain detectable at the ZIP-quarter level.

## Discussion

This study provides evidence of a positive causal relationship between power outages and mental health hospitalizations, using de-identified discharge-level administrative data from Maryland (2018–2023) and multiple identification strategies. Although our estimates are specific to Maryland, the findings raise a broader hypothesis that recurrent power outages may contribute to mental-health burdens in other regions with similar grid vulnerabilities, climate-related outage risks, and healthcare-system constraints. Globally, mental disorders affected an estimated 970 million individuals in 2019 and remained among the top ten leading causes of disease burden.^47^ The full scale of this burden is likely underestimated: when premature mortality attributable to mental disorders is included alongside morbidity, an estimated 418 million disability-adjusted life years (DALYs)—approximately 16% of all global DALYs—can be attributed to mental disorders, with an associated economic cost of approximately USD 5 trillion.^48^ In the US., more than 22% of the adult population is affected by mental health disorders,^24^ and economic losses attributable to mental disorders in high-income North America could account for up to 8% of gross domestic product.^48^

Maryland presents a particularly acute case: its mental health service utilization rate (42.2 per 1,000 population) is nearly twice the national average (24.5 per 1,000),^25^ indicating an already-elevated baseline demand for psychiatric care that additional outage-related hospitalizations may further strain. Aging infrastructure and increasingly frequent climate-related extremes place energy systems worldwide at growing risk of major outages.^49^^,^^50^ Baseline PPML estimates indicate a modest increase in hospitalization rates associated with outage exposure, while IV estimates confirm a positive and statistically significant effect. Across specifications, the results are consistent in direction. Sensitivity analyses further show that the effect is driven primarily by short-duration outages (1–3 h)—frequent disruptions that characterize everyday grid operations—rather than rare, prolonged blackout events.

These findings contribute to a growing literature linking power outages to adverse health outcomes. Prior work has documented mortality effects and mental health impacts during major disasters. However, existing literature largely focuses on extreme events and often cannot isolate the independent effect of outages from concurrent shocks. By contrast, our results provide causal evidence that even routine, everyday outages are associated with increases in hospitalizations for primary mental health conditions.

The stronger and more stable associations observed for 1–3h outages might partly reflect the greater frequency of these events and therefore greater statistical variation, rather than evidence that longer outages are harmless. The heterogeneity results also suggest that hospitalization-based estimates reflect both underlying need and access to care. Associations are more readily detected in urban ZIP codes, lower-poverty areas, and communities with greater Medicaid-related inpatient use. This pattern is consistent with the possibility that outage-related distress is more likely to be captured in hospital records where psychiatric care, referral pathways, and transportation are more accessible. Smaller or null hospitalization effects in underserved areas should therefore be interpreted cautiously, because they might reflect lower access to inpatient care, delayed care, or distress that remains outside formal hospital systems rather than an absence of mental health burden.

These findings could have implications for infrastructure planning and regulatory decision-making. Standard reliability metrics and cost-benefit analyses usually emphasize engineering performance, outage duration, or customer interruption costs; they rarely include mental health service use, household coping costs, or public-payer spending. If outages contribute to psychiatric hospitalizations, current planning frameworks might undervalue the broader health relevance of reliability investments.

Policy discussions should therefore distinguish between rare catastrophic blackouts and recurrent moderate disruptions. Major prolonged events remain medically important, especially for people dependent on powered medical equipment. At the same time, our results suggest that routine 1–3 h interruptions warrant attention because they are frequent and often receive less formal emergency response. Reliability planning that accounts for repeated short interruptions could better capture health-relevant dimensions of grid performance.

From a policy perspective, the key implication is prioritization rather than a single universal intervention. Utilities, regulators, emergency managers, and health agencies could combine outage histories with indicators of mental health burden, Medicaid reliance, electricity-dependent medical needs, and limited care access to identify communities where reliability investments and outage-period outreach might be most important. Outage preparedness could include measures to support continuity of mental health care, such as backup options for telehealth, pharmacy refills, medication storage, crisis-line capacity, transportation to urgent care, and communication with high-risk patients during interruptions.

Looking forward, the relevance of these findings could increase as electricity systems face rising demand and more frequent climate-related stressors. Electrification of transportation and heating, growth in data-center demand, and aging infrastructure could increase pressure on grid reliability at the same time that electricity becomes increasingly important for routine care delivery and household health protection. More broadly, these results support considering grid reliability not only as an engineering or economic issue, but also as a public health planning issue. Linking outage feeds with health-system data could help monitor emerging risks, evaluate reliability investments, and target preventive outreach, although future research is needed to test whether such strategies reduce outage-related health burdens.

### Limitations of the study

Several limitations should be noted. First, our outcome measure captures only inpatient admissions with a primary mental health diagnosis, and therefore reflects only a subset of individuals who access formal psychiatric care. Second, the analysis is limited to Maryland and may not generalize to regions with different infrastructure, climate conditions, or healthcare systems. Third, the quarterly resolution of the hospitalization data prevents assessment of short-term lag structures after outage events. Fourth, the IV estimates identify local average treatment effects and do not allow us to distinguish among specific behavioral, clinical, or service-disruption pathways linking outages to mental health outcomes.

## Resource availability

### Lead contact

Requests for further information and resources should be directed to and will be fulfilled by the lead contact, Yueming (Lucy) Qiu (yqiu16@umd.edu).

### Materials availability

This study did not generate new unique reagents or materials.

### Data and code availability

• Data: The de-identified discharge-level records from the Healthcare Cost and Utilization Project State Inpatient Databases (HCUP SID), Maryland, 2018–2023, are subject to the HCUP Data Use Agreement and cannot be redistributed by the authors. Access may be requested through the HCUP Central Distributor. The historical Maryland power outage records used in this study were obtained directly from the State of Maryland Department of Information Technology through a separate data request. The historical records cover 2014–2023, and records from 2018 to 2023 were used in the health-outcome analysis. These data are not publicly available and cannot be redistributed by the authors; researchers seeking access should contact the data provider directly. American Community Survey, Area Health Resources Files, and NOAA GHCN-Daily data are publicly available from the sources listed in the key resources table.
• Code: The Stata replication code underlying the reported statistical analyses, tables, and Stata-generated figures has been deposited at Zenodo and is publicly available at https://doi.org/10.5281/zenodo.22119931. Python was used for preprocessing restricted source data, and Figures 4 and 5 were produced in ArcGIS Pro.
• Additional information: Any additional information required to reanalyze the data reported in this paper is available from the lead contact upon request.

## Acknowledgments

The authors gratefully acknowledge the data support, with power outage data for Maryland credited as: Courtesy of the State of Maryland, Department of Information Technology. Jie Chen was supported by the National Institute on Aging (grants R01AG062315, RF1AG083175, and P30AG097158). Meng Feng was supported by a fellowship from the University of Maryland Institute for Public Leadership.

## Author contributions

Conceptualization, M.F., Y.Q., and J.C.; methodology, M.F.; formal analysis, M.F.; investigation, M.F.; data curation, M.F.; visualization, M.F.; writing – original draft, M.F.; writing – review and editing, M.F., Y.Q., J.C., J.L., and Y.D.W.

## Declaration of interests

The authors declare no competing interests.

## STAR★Methods

### Key resources table

**Table:** 

REAGENT or RESOURCE	SOURCE	IDENTIFIER
**Depositeddata**		

HCUP State Inpatient Databases (SID), Maryland, 2018–2023	Agency for Healthcare Research and Quality (AHRQ)	https://hcup-us.ahrq.gov (restricted access)
Historical Maryland power outage records (15-min interval, utility-provider level), 2014–2023	State of Maryland, Department of Information Technology	Restricted access; obtained through a separate data request; access subject to approval by the data provider
Socioeconomic characteristics (ACS)	US. Census Bureau	https://data.census.gov/
Area Health Resources Files (AHRF)	Health Resources and Services Administration	https://data.hrsa.gov/topics/health-workforce/nchwa/ahrf
Weather data	NOAA, via Climate Data Online (GHCN-Daily)	https://www.ncdc.noaa.gov/cdo-web/

**Softwareandalgorithms**		

Replication code for the statistical analyses	This paper	Zenodo: https://doi.org/10.5281/zenodo.22119931
Stata 18 MP	StataCorp	https://www.stata.com
Python	Python Software Foundation	https://www.python.org
ArcGIS Pro	Esri	https://www.esri.com/en-us/arcgis/products/arcgis-pro

### Experimental model and study participant details

This study was conducted under a University of Maryland, College Park Institutional Review Board protocol (IRBNet no. 931655-12; internal reference no. 00032768; principal investigator: Jie Chen). The February 18, 2025 amendment/modification was acknowledged by the UMCP IRB and added Meng Feng to the project team. The analysis used de-identified secondary administrative records from the HCUP State Inpatient Databases.

### Method details

#### Data

This study combines data from multiple sources to analyze the relationship between power outages and mental health-related hospital and emergency visits in Maryland. The primary dataset includes administrative hospital discharge data from the Healthcare Cost and Utilization Project (HCUP), specifically the State Inpatient Database (SID) for Maryland, covering the years 2018–2023. We focus on visit-level (per discharge level) records rather than patient-level data. HCUP does not allow tracking individual patients over time, which means longitudinal analysis at the individual level is not feasible. Furthermore, HCUP SID database captures only hospital-based encounters— inpatient hospitalizations—but does not include office-based care, such as visits to outpatient clinics, psychologists, or primary care physicians. As a result, this study likely underrepresents mild-to-moderate mental health cases that do not lead to hospital visits.

#### Defining mental health outcomes

Our primary outcome is inpatient admissions with a primary mental health diagnosis, identified in HCUP SID database using the principal diagnosis only (DX1), so that mental health is the main reason for hospitalization rather than a comorbidity. We classify diagnoses using ICD-10 mental health codes (refer to Method S1 for the details) and aggregate discharge records to the ZIP code-quarter level for Maryland (2018–2023). For each ZIP code-quarter, we construct “mh_primary_cases”, defined as the count of admissions with a primary mental health diagnosis. The full list of ICD-10-CM principal-diagnosis codes used to construct this indicator is provided in Table S1, and the prevalence of mental health conditions by diagnosis type is summarized in Table S3.

The HCUP SID Maryland file (2018–2023) includes de-identified patient discharges with ZIPCODE identifiers and provides details such as age, gender, race/ethnicity, primary and secondary diagnoses, and the patient’s county. The HCUP SID does not include exact discharge dates; temporal information is limited to discharge month (DMONTH) and quarter (DQTR). For Maryland, only DQTR is available.^51^ Consequently, hospital discharge records are aggregated to the ZIP code–quarter level.

The sample includes 3,075,969 inpatient cases for Maryland residents (2018–2023). Of these, 194,840 (6.33%) had a mental health primary diagnosis, while 1,106,228 (35.96%) had mental health conditions only as secondary diagnoses, indicating that mental health is more often recorded as a comorbidity than the main reason for admission. Across any diagnosis position, substance use disorders were most common (19.61%), followed by depression (18.95%), anxiety (17.07%), psychotic disorders (2.90%), and stress-related disorders (2.62%); categories are not mutually exclusive. The main analysis uses primary-diagnosis mental health admissions only, which improves specificity but likely underestimates true ZIP code-level burden because inpatient data exclude undiagnosed and outpatient-only cases.^52^

#### Mental health as a secondary diagnosis

To capture admissions in which a mental health condition co-occurs with a physical principal diagnosis, we additionally flag ICD-10-CM mental health codes (F00–F99) in the secondary diagnosis fields (I10_DX2–I10_DX101). We define mh_secondary_cases as the number of admissions in each ZIP code-quarter with a non-mental-health principal diagnosis and at least one mental health code in a secondary position, and nonmh_nocomorb_cases as the number of admissions with no mental health code in any diagnosis field, which serves as a contrast group. For the vulnerable-population analysis, we identify five groups by principal diagnosis—diabetes (E08–E13), COPD and asthma (J40–J47), heart disease (I20–I25 and I50), dementia (G30–G31), and chronic kidney disease (N18)—and split each group by the presence of coexisting depression or anxiety (F32, F33, F41, or F43) in the secondary diagnosis fields. Dementia is defined using G30–G31 only, because vascular and other dementias coded F01–F03 fall within the mental health definition. All secondary-diagnosis outcomes are estimated with the same specification as the main results, except that we exclude the two Elixhauser comorbidity-index controls because these controls are constructed from the same secondary diagnosis fields as the outcomes.

#### Power outage (PO) data

We reviewed prior studies to summarize how power outages have been defined in the literature using US.-based outage data. Most studies construct a binary exposure variable based on percentile thresholds of the proportion of customers affected by outages in a given geographic area. For example,^40^^,^^41^^,^^42^^,^^43^ all classify a division-day as a PO day if PO coverage exceeds the 50^th^ percentile of its distribution, while^44^ apply the 75^th^ percentile. In these studies, each day meeting the threshold is classified into mutually exclusive day types (e.g., PO only, hazard only, PO + hazard, neither) for use in daily time-series models. However, because our analysis is conducted at the quarterly level, a binary indicator would obscure meaningful variation: nearly all ZIP-quarters in our sample experience at least one outage, so a binary measure cannot distinguish a ZIP-quarter with one episode from another with dozens. We therefore use episode counts as a continuous exposure measure to capture the intensive margin of repeated disruption.

Our episode identification procedure draws on Do et al. (2023), who used data from PowerOutage.us at 10-min, county-level resolution and defined outage events as continuous periods in which the proportion of customers without power met or exceeded 0.1% (the 90^th^ percentile of county-hourly outage rates). They classified events by duration, distinguishing 1+hour outages (which disrupt commerce and daily routines) and 8+ hour outages (a “medically relevant” duration exceeding the battery life of most electricity-dependent medical equipment and long enough for food, medication, and thermal regulation to be compromised).

PO records at the utility provider level were obtained from the Maryland State Department, recorded every 15 min from 2018 to 2023. Each record includes the timestamp of the PO, the provider, and the number of customers affected within each ZIP code in Maryland. Because many utilities do not report the total number of customers served at the ZIP level, we adopt the approach used by poweroutage.us^18^ to approximate customer coverage. In this approach, “Customers Tracked” for each ZIP–provider pair is defined as the maximum number of customers affected observed historically (2014–2023), which is treated as a proxy for the total customer base in that area (see Method S2 for details). PO coverage is then calculated as the ratio of customers affected to the estimated total number of customers tracked:

$${\;\text{OutageRate}\;}_{i}=\frac{{\;\text{CustomersAffected}\;}_{i}}{{\;\text{CustomersTracked}\;}_{\text{zip},\text{provider}\;}}$$

where Customers Tracked represents the maximum observed outage for each ZIP code-provider combination across the entire study period. A PO occurrence (Yes/No) is then defined as any 15-min interval in which PO coverage exceeded the 75th percentile of the distribution among all intervals with nonzero outages (0.51% of customers affected at any given time point; such as the 75^th^ percentile). This threshold focuses on severe, large-scale events and reduces noise from minor disruptions, consistent with prior power outage definitions in the literature summarized in Table 1). Applying this criterion yields 2,003,452 observations (16.54% of total records) classified as PO occurrences and 151,276 distinct outage episodes identified across 1,191 ZIP codes.

To identify distinct outage events, consecutive flagged observations are grouped into continuous episodes, where observations separated by 25 min or less were considered part of the same episode (see Method S2 for the episode identification details). Our primary exposure variable in the baseline specification, “new_1hr_episodes_p75,” is defined as the number of outage episodes in ZIP code *i* and quarter *t* that last at least 1 h and exceed the 75th percentile of outage intensity within the sample. The threshold (p75) indicates that only the most severe 25% of outage events—those with an outage rate above the 75th percentile—are included. Episodes are assigned to the quarter in which they begin, so that outage events spanning multiple quarters are counted only once rather than being double-counted. We construct the log-transformed measure (to normalize the right-skewed distribution and allows coefficients to be interpreted as approximate percentage changes) (Method S2 for detailed calculation of the power outage variable):

$$\ln\;{\;\text{outage}\;}_{\text{it}}=\ln\left(\;\text{new}\_1\text{hr}\_\text{episodes}\_p\;75_{\text{it}}+1\right)$$

where new_1hr_episodes_p75 ${\;}_{\text{it}}$ denotes the number of 1-h outage episodes at the 75th percentile threshold for ZIP code i in quarter t. The percentile-based severity threshold follows the power outage-health literature,^40^^,^^41^^,^^42^^,^^43^^,^^44^ while the episode identification procedure-flagging continuous periods where outage severity exceeds a percentile cutoff and persists beyond a minimum duration-parallels the methodology in Do et al. 2023. The log transformation compresses the right-skewed distribution while the constant 1 is added to accommodate zero values. Results are robust to using untransformed episode counts in place of the log-transformed measure (IRR = 1.002, *p* < 0.10; Full results are reported in Table S14), suggesting that the positive association does not depend on the functional form of the exposure variable. The magnitude is consistent with the log-transformed specification: a ZIP code moving from the 25th to the 75th percentile of outage exposure—approximately 6 additional episodes per quarter—would see a 1.2% increase in primary mental health hospitalization risk (1.002^6^ ≈ 1.012), comparable to the baseline estimate from the log specification (IRR ≈1.015–1.017). A detailed description of the variable construction procedure is provided in Method S2.

Due to data limitations, admission dates in the HCUP Maryland SID database are only available at the quarterly level, while power outage data are available at 15-min intervals. We therefore aggregate both datasets to the ZIP code-quarter level for analysis in Maryland. The final dataset contains quarterly counts of outage episodes for each ZIP code, along with additional measures including episode counts at alternative duration thresholds (2–8 h) for sensitivity and robustness checks.

#### Control variables and integration with external data

To improve causal inference and control for confounding factors, we integrate several external datasets with the HCUP Maryland SID.

**Socioeconomic** **characteristics** – including total population, poverty rate, unemployment rate, and median household income – were obtained from the five-year estimates of the US. Census Bureau’s American Community Survey (ACS) at the ZIP Code Tabulation Area (ZCTA) level. These variables capture community-level economic vulnerability that may influence both infrastructure reliability and healthcare utilization patterns.

**Healthcare** **access** **variables** were extracted from the Area Health Resources Files (AHRF), including psychiatrist supply per capita, hospital bed capacity, and primary care physician availability (see Method S4 for detailed variable definitions). These controls account for geographic variation in healthcare resources that may affect mental health service utilization independent of power outage exposure.

**Discharge-level** **clinical characteristics** were aggregated from the HCUP Maryland SID to the ZIP-quarter level to control for underlying population health status. We include the Elixhauser Comorbidity Index to adjust for physical health conditions that may mediate outage effects (e.g., diabetes patients requiring insulin refrigeration, cardiac patients relying on medical devices), as well as baseline mental health burden measured by the prevalence of psychoses, alcohol-related disorders, and drug abuse (see Method S5 for comorbidity index construction).

**Weather variables** were incorporated to account for environmental factors that may jointly influence both power outages and mental health outcomes. We include measures of extreme heat days, extreme cold days, precipitation, and wind conditions as controls. These variables isolate the effect of power outages from the direct physiological and psychological impacts of adverse weather (see Method S3 for weather variable construction and threshold definitions). The specific percentile-based thresholds for these extreme-weather variables are reported in Table S4.

The integration of these datasets allows us to control for environmental, socioeconomic, healthcare access, and clinical factors that may confound the relationship between power outages and mental health outcomes.

### Quantification and statistical analysis

#### Empirical strategy

We estimate the effect of power outage exposure on mental health admissions using Poisson Pseudo-Maximum Likelihood (PPML). This approach is well-suited for non-negative count outcomes, robust to overdispersion, and does not require strict distributional assumptions. The model includes total ZIP-quarter population as the exposure term, estimating per-capita admission rates.

We adopt the Poisson Pseudo-Maximum Likelihood (PPML) estimator rather than a log-linear OLS specification (e.g., reghdfe). This approach is well-established in the health economics literature for modeling hospitalization counts using administrative data.^53^^,^^54^ employ PPML to model hospital inpatient utilization rates and service demand following health care reform using the HCUP SID/SEDD database, with population as the exposure term—the same specification we adopt. More recently,^55^ use a Poisson count data model—numerically equivalent to PPML—to estimate the effect of air pollution on influenza hospitalizations using HCUP administrative records at the county-month level. Our empirical setting closely mirrors these studies: all three use HCUP administrative hospitalization records aggregated to geographic-by-time units, with non-negative count outcomes and population-based exposure terms. Following the methodological rationale established in these studies, PPML is preferred over log-linear OLS for several reasons. First, as^55^ note, “the PPML estimator performs well with a large number of zeros and over-or-underdispersion in the data”—both features present in our data, where many ZIP-quarters record zero mental health hospitalizations. A log-linear OLS approach would require transforming the outcome (e.g., log(y + 1)), which discards information from zeros and introduces bias. Second, the PPML estimator “yields consistent parameter estimates regardless of whether the dependent variable follows a Poisson distribution, so long as the conditional mean is properly specified,” and “the consistency properties of the estimator are retained” even when the variance assumption is violated.^53^ This is particularly relevant in our setting, where hospitalization counts vary substantially across ZIP codes of different population sizes. By incorporating total ZIP-quarter population as an offset, our PPML specification directly estimates per-capita admission rates, making the coefficients interpretable as incidence rate ratios.

The baseline specification adopts a two-way fixed effects regression. ZIP code fixed effects account for time-invariant local characteristics such as geography, infrastructure, and demographic composition. Year-quarter fixed effects absorb shocks common to all areas, including seasonal trends and statewide policy or economic shifts. In other words, we assume that within a given ZIP code, any change in the frequency or intensity of outages over time is uncorrelated with unobserved determinants of mental health admissions in that area, after accounting for controls.

We include several observed time-varying covariates to further mitigate concerns about confounding. These include: socioeconomic and demographic factors (poverty rate, median household income, percent male, percent Black, percent Asian); insurance coverage (Medicaid share, uninsured share); healthcare supply (psychiatrists per 100,000 residents); and health vulnerability measures (case-mix-adjusted comorbidity and mortality indices). By including these covariates, we address the possibility that outage exposure may be correlated with underlying trends in local health vulnerability, access to care, or socioeconomic stressors that also influence mental health outcomes. Summary statistics for all model variables are reported in Table S5, and pairwise correlations and multicollinearity diagnostics (variance inflation factors) are provided in Tables S6–S8.

The outcome is measured at the ZIP code-by-quarter level. Because we include total ZIP-quarter population as an offset (exposure) term, the PPML model estimates the admission rate per capita rather than raw counts. This makes the interpretation of coefficients comparable to rate-based outcomes often used in health services research.^56^^,^^57^

Formally, the model is specified as:

$$\begin{array}{c} \;E\left[{\;\text{mh}\_\text{cases}\;}_{\text{iqy}}\right]=\exp(\ln\left({\;\text{population}\;}_{\text{iqy}}\right)+\beta_{1}\;\ln\left(1+{\;\text{outage}\;}_{\text{iqy}}\right)+\beta_{2}{\;\text{heat}\;}_{\text{iqy}}+X_{\text{iqy}}^{\prime }\gamma +\delta_{\text{qy}}+\theta_{i}) \end{array}$$

where:

• mh_cases_iqy_denotes the number of primary mental health hospitalizations in ZIP *i*, quarter *q*, year *y*;
• population_iqy_is the total population used as an exposure term;
• *ln*⁡(1+outage_iqy_)is the log-transformed count of significant outage episodes, using *ln*⁡(episodes+1)to address the zero-inflated and right-skewed nature of the data;
• heat_iqy_is the number of extreme heat days (days above the ZIP-specific 90th percentile);
• X_iqy_is the vector of time-varying control variables;
• δ_qy_are year-by-quarter fixed effects;
• θ_i_are ZIP code fixed effects.

Standard errors are clustered at the ZIP code level to account for serial dependence within areas over time. We report incidence rate ratios (IRRs) rather than log coefficients to improve interpretability: an IRR greater than 1 indicates an increase in admission rates associated with the explanatory variable, while an IRR less than 1 indicates a reduction. For example, an IRR of 1.20 on implies that a one-unit increase in logged outage episodes is associated with a 20% increase in the rate of mental health admissions, holding other variables constant.

#### Instrumental variable strategy

Although the baseline PPML regressions include ZIP code fixed effects, year-quarter fixed effects, and time-varying controls, unobserved factors correlated with both outage exposure and mental health hospitalization could still bias estimates. To address endogeneity concerns more directly, we implement an instrumental variable (IV) approach using electricity infrastructure density – substation density, transmission line density and transformer density–as instruments for power outage exposure (see Method S6 for details on construction of the instrumental variables).

The electric power grid operates as a hierarchical system with distinct transmission and distribution layers (see Note S3). This architecture creates two vulnerability pathways that we exploit for identification. Failures in the upstream transmission network (substations and high-voltage lines) tend to produce widespread, cascading outages affecting large geographic areas, whereas failures in the downstream distribution network (transformers and distribution lines) typically generate more frequent but geographically localized disruptions. To capture these mechanisms, we construct instruments reflecting vulnerabilities at different grid layers. Transformer density measures the structure of the local distribution network serving end users, while substation density and high-voltage transmission line density capture exposure to disturbances in the regional bulk power system. The spatial distribution of these infrastructures varies substantially across Maryland ZIP codes (Figures 4 and 5) and is largely determined by long-term engineering, geographic, and regulatory considerations rather than short-run local health conditions (see Note S3).

A limitation of the IV approach is that electrical grid infrastructure is inherently slow-changing capital stock. The instruments are therefore time-invariant and cannot be combined with ZIP code fixed effects. Our IV specification instead relies on cross-sectional identifying variation, controlling for time trends through year-quarter fixed effects and for ZIP-level confounders through a rich set of covariates.

## Supplemental information

Document S1. Figures S1, Tables S1–S17, Methods S1–S6, and Notes S1–S4
