# Supplementary material for: Recurrent power outages and the mental health burden of grid reliability in Maryland
Source: iScience. 2026 Sep 16;29(10):117513. doi: 10.1016/j.isci.2026.117513 (PMC13599647; doi:10.1016/j.isci.2026.117513)
Supplement: Supplementary file 1 [file mmc1.pdf]

iScience, Volume *29*

Supplemental information

Recurrent power outages and the mental health burden of grid reliability in Maryland

Meng Feng, Yueming (Lucy) Qiu, Jie Chen, Jiehong Lou, and Yi David Wang

Supplemental information

Note S1. Literature review on Power outage, extreme weather and Mental Health, Related to Introduction

Literature on extreme weather events, power outages, and mental health

Prior research has documented that severe weather events and power outages frequently co-occur at the same time in many U.S. regions. [S1] found that 62.1% of medically relevant (8+ hour) power outages coincided with extreme weather or climate events, with outages 3.4 times more common on days with an isolated severe weather event and 10 times more common on days with multiple simultaneous events. In a follow-up study, [S2] further characterized these co-occurrence patterns across 1,657 U.S. counties, showing that extreme precipitation was the most common weather type co-occurring with 8+ hour outages (affecting 70.6% of study counties), followed by extreme heat (50.6%), and that the conditional probability of an outage was highest during tropical cyclones (0.24) and lowest during periods of extreme cold (0.01). This pervasive co-occurrence of outages and severe weather means that individuals experiencing power loss are often simultaneously exposed to extreme temperatures, storms, or other climate stressors.

Climate-related hazards of various forms – such as hurricanes, floods, wildfires; subacute stressors such as heatwaves and droughts; and long-lasting environmental changes such as rising temperatures and sea levels- are linked to elevated risks of anxiety, depression, post-traumatic stress disorder, suicide, and other adverse psychological outcomes [S3-S5]. Extreme weather and power outages are related but distinct stressors that affect health through different pathways. Extreme weather—especially high temperatures—can directly worsen mental health through physiological and behavioural channels, including thermoregulatory stress, sleep disruption, irritability, and cognitive impairment [S6-S9]. Individuals with pre-existing mental illness are particularly vulnerable. Psychotropic medications can impair the body's ability to regulate internal temperature under extreme heat, thereby increasing physiological risk among patients [S10,S11], and individuals with mental illness may be less likely to recognize heat-related risks or take appropriate protective actions [S37]. A systematic review of 22 studies further confirms that individuals with mental illness consistently experience worse morbidity and mortality outcomes during high-temperature days compared to those without mental illness, although evidence is more mixed for multi-day heatwaves [S12] (refer to Supplementary Note S1 for more details).

Power outages may influence mental health through infrastructure-dependent pathways that are distinct from direct weather effects. Evidence suggests that outage-related disruptions to cooling or heating systems (e.g., thermal discomfort due to inability to control the indoor environment, sleep disruption related to heat and cold exposure) can

further intensify psychological distress, especially during extreme weather conditions, although most existing studies identify this mechanism in the context of co-occurring hazards rather than in isolation [S13,S14]. Beyond physical discomfort, outages may generate psychological distress through disrupted daily routines, uncertainty about restoration timing, and reduced access to social support [S13,S15].

For people with pre-existing psychiatric conditions, outages may further destabilize symptoms by disrupting treatment continuity (telehealth access, medication refills, crisis support), especially during cascading events [S16,S17]. These risks are socially patterned: vulnerable groups include older adults, people with psychiatric conditions, and those dependent on electrically powered medical equipment [S15,S18], and ZIP-level evidence suggests higher outage frequency in lower-income communities and shorter durations where critical facilities are present [S19]. This inequality pattern is consistent with broader reviews documenting disproportionate outage burdens among socioeconomically vulnerable, racial/ethnic minority, and rural populations [S20]. Although prior administrative studies have focused mainly on non-psychiatric outcomes, they show clinically meaningful healthcare utilization responses (elevated emergency department visits) to outages [S21], supporting the plausibility of outage-related mental health impacts in settings with existing treatment gaps and social vulnerability gradients [S22,S23]. The importance of stable energy access for psychological well-being is underscored by evidence from Germany, where energy-poor households – those unable to keep homes adequately warm due to financial constraints – had significantly worse self-rated health and mental-health outcomes [S24]. Together, these findings highlight that power supply disruptions can affect mental health through multiple pathways; yet the evidence remains largely anchored to major disasters and catastrophic events, leaving the mental health consequences of routine, non-catastrophic outages at the population level empirically unexamined — a gap this study directly addresses.

While both weather and outage pathways are supported by existing evidence, prior empirical studies have generally been unable to separate their independent contribution to mental health outcomes because these stressors co-occur during disasters. In a study of Hurricane Sandy, [S25] found that each 1-percentage-point increase in the maximum daily percentage of households experiencing blackouts in the Bronx was associated with an 8.82-fold increase in mental health ED visit prevalence – but because the hurricane simultaneously caused flooding, property destruction, displacement, and water service disruptions, it is unclear whether the observed mental health effects reflect the power outage, the trauma of the storm itself, or the loss of other essential services. Similarly, [S26] found that Texas residents who experienced both power and water outages during Winter Storm Uri, combined with high Covid-19 impacts, had 7.7 times higher odds of post-traumatic stress – yet the joint occurrence of extreme cold, power loss, water loss, and pandemic stress makes it difficult to isolate the contribution of any

single factor. This identification challenge extends to physical health outcomes: [S27] found that the combined exposure to windstorms and power outages produced the highest increase in injury-related ED visits in New York State, while power outages alone did not significantly affect injury visits, suggesting that the health effects observed during disasters may be driven primarily by the weather event rather than the outage. [S28] compared nursing home residents in facilities that lost power with those that maintained power during Hurricane Irma, and found that power loss was associated with 25% higher odds of mortality within 7 days and 12% higher odds within 30 days. [S29] reported a similar pattern for cardiovascular and respiratory hospitalizations during winter storms: the joint exposure category (storm plus outage) consistently yielded the strongest effects, while the independent contribution of outages was less clear.

Several natural hazard–power outage studies classify exposure into four mutually exclusive day types: hazard-only, outage-only, hazard+outage, and non-event (reference) days. In New York, [S27] found the highest injury ED risk on joint wind+outage days (RR (relative risk) = 1.14, means the outcome risk is 14% higher in the exposed group vs. reference), while outage-only days were not statistically significant; all estimates were relative to non-event days (days with neither a wind event nor an outage). Using a similar day-type framework, [S29] examined winter storms and outages (with outage defined as coverage above the 50th percentile among operating divisions with outages) and likewise found the strongest effects under joint exposure, including a 25% increase in cardiovascular hospitalizations (RR = 1.25) and a 54% increase in lower respiratory disease hospitalizations (RR = 1.54), both relative to non-event (non-storm/non-outage) days. Outage-only effects were more limited and delayed (e.g., injuries +22% at lag day 1; food/water-borne diseases +51% at lag day 3), with an unexpected decrease in lower respiratory disease at lag day 5 (RR = 0.88, lower risk, 12% lower). These studies are useful because they separate hazard-only, outage-only, and hazard+outage days. They show that the largest health risk often appears on joint-exposure days, but it does not by itself test whether the joint effect is greater than the sum (additive interaction) or product (multiplicative interaction) of the two single effects.

Note S2. IV Robustness Checks: Alternative Outage Definitions, Related to STAR Methods

Figure S1 presents IV second-stage estimates across seven alternative episode-based definitions of power outage exposure, ranging from episodes lasting at least 8 hours (P50 severity) to the baseline measure of at least 1 hour (P75 severity). The results

demonstrate that the positive association between outages and mental health hospitalizations is robust, and that shorter-duration, higher-frequency outage measures yield more precise estimates.

Specifications (a) through (g) progress from longer to shorter minimum duration thresholds. Specifications (a) and (b), using episodes lasting at least 8 hours and 6 hours above the 50th percentile of severity, yield large but statistically imprecise coefficients of 1.226 and 0.863, respectively, reflecting the rarity of such prolonged events and the corresponding weakness of the first stage ($F = 7.89$ and 8.95). Specification (c), using episodes lasting at least 5 hours (P50 severity), yields a coefficient of 0.697 ($p < 0.10$; IRR = 2.01). Specification (d), using episodes lasting at least 4 hours, yields 0.555 ($p < 0.10$; IRR = 1.74). Specification (e), using episodes lasting at least 3 hours above the 50th percentile, produces a more precisely estimated effect of 0.482 ($p < 0.05$; IRR = 1.62). Specifications (f) and (g) move to the 75th percentile severity threshold: specification (f), restricting to episodes lasting at least 2 hours, yields 0.416 ($p < 0.01$; IRR = 1.52), while specification (g)—the baseline measure of at least 1 hour (P75)—yields the most precisely estimated effect of 0.380 ($p < 0.01$; IRR = 1.46).

The pattern across specifications is clear and interpretable: as the minimum duration threshold decreases, the estimates become more statistically precise while the point estimates decline in magnitude, reflecting increased instrument strength and greater episode frequency. First-stage F-statistics decline monotonically from 19.76 for the baseline measure (≥1hr P75) to 7.89 for the longest-duration specification (≥8hr P50), confirming that shorter-duration outage measures are more strongly predicted by grid infrastructure density. The declining precision at longer duration thresholds reinforces the interpretation that moderate-duration outages—which are sufficiently frequent to generate meaningful instrument variation—drive the strongest and most reliable associations with mental health outcomes.

Across all seven specifications, the IV estimates remain positive, with statistically significant effects observed in specifications (c) through (g) (IRR = 1.46–2.01). All Hansen J overidentification tests pass (p-values range from 0.399 to 0.948), confirming instrument validity across alternative outage definitions. The consistency of results across different episode-based operationalizations—using varying duration and severity thresholds—strengthens confidence that the estimated effects reflect a causal relationship between power outages and mental health outcomes.

Note S3. Instrumental Variable Strategy, Related to STAR Methods

The electric power grid operates as a hierarchical system connecting generation facilities to end users through distinct network layers [S30]. Electricity flows from generation stations (approximately 20kV) through step-up transformers to high-voltage transmissions lines (69-765kV), then through substations and step-down transformers to distribution networks. The distribution system consists of primary lines (5-35kV) and secondary lines (115/230V) that deliver power to individual meters and circuit breakers at end-user premises.

We construct instrument capturing vulnerabilities at different grid layers:

(1) Transformer density (local distribution infrastructure)

We use transformer density as an instrument capturing the structure of the local, lower-voltage distribution network that delivers electricity directly to end users. Areas with higher transformer density typically have more robust and redundant distribution networks, which reduces the likelihood and duration of service interruptions. Conversely, areas with sparse transformer coverage may experience more frequent outages due to limited redundancy and longer repair distances. Consistent with this mechanism, the first-stage results show a negative relationship between transformer density and outage exposure.

The exclusion restriction requires that transformer density affects mental health outcomes only through its effect on power outages. This assumption is plausible because the placement and density of transformers are primarily determined by long-term infrastructure planning decisions, engineering requirements related to load demand, and geographic considerations, rather than by short-run neighbourhood socioeconomic conditions or health status. To further mitigate potential confounding, our empirical models control for a rich set of demographic, socioeconomic, and health-related covariates.

(2) Upstream transmission infrastructure (system-level exposure)

We also use two alternative instrumental variables capturing exposure to the upstream transmission network: substation density and high-voltage transmission line density. These variables characterize the structure of the regional bulk power system. Areas with a higher density of substations and high-voltage transmission lines tend to be more deeply integrated into the transmission network and often function as major load or transfer nodes. During extreme weather events or grid disturbances, failures in such highly interconnected systems are more likely to propagate across large geographic areas, generating widespread and prolonged outages. The spatial configuration of transmission infrastructure is largely determined by long-term engineering requirements, geographic constraints, and regulatory planning rather than by short-run local health conditions.

The exclusion restriction requires that grid infrastructure affects mental health hospitalizations only through its impact on outage exposure. Several features of the empirical design support this assumption. First, transmission infrastructure is highly persistent and evolves slowly over time. Substations and transmission lines are sited based on engineering requirements, rights-of-way, and long-term load forecasts, and therefore do not respond to short-run fluctuations in local health outcomes [S30]. Identification thus relies on cross-sectional variation in infrastructure interacting with time-varying shocks rather than on contemporaneous infrastructure investment.

Second, the IV specifications include year-by-quarter fixed effects, which absorb time-specific shocks common to all locations, including seasonal patterns, statewide policy changes, and macroeconomic conditions. Third, the models control for a comprehensive set of time-varying local characteristics—including socioeconomic conditions, insurance coverage, healthcare supply, baseline health vulnerability indices, and extreme weather exposure—thereby reducing the possibility that infrastructure density proxies for contemporaneous changes in community vulnerability or healthcare access. Although infrastructure placement may correlate with long-run characteristics such as population density, these factors are directly controlled for in the regressions. The remaining variation therefore reflects the physical configuration of the electricity grid, which determines how outages propagate through the network but does not directly influence psychiatric hospitalization demand.

Note S4. Mental health as a secondary (comorbid) diagnosis: construction and results, Related to STAR Methods

Motivation: Our primary outcome counts admissions in which a mental health condition is the principal diagnosis (DX1), that is, the main reason for the hospitalization. This definition does not capture admissions in which a mental health condition contributed to, or co-occurred with, a physical condition: in such cases the physical condition is recorded as the principal diagnosis and the mental health condition appears in a secondary position. Because the Maryland SID records up to 101 diagnosis codes per admission, these compound cases can be identified directly from the data.
Construction. For each of the 3,075,969 Maryland inpatient records (2018–2023), we flag any ICD-10-CM mental health code (F00–F99) in the secondary diagnosis fields (I10_DX2–I10_DX101). We then count, for each ZIP code-quarter: (1) admissions with a non-mental-health principal diagnosis and at least one mental health code in a secondary position (mh_secondary_cases); and (2) admissions with no mental health code in any diagnosis field (nonmh_nocomorb_cases), which serve as a contrast group. Mental health comorbidity is common: 38.4% of admissions without a primary mental health diagnosis carry a mental health code in a secondary position (1,106,228 cases), compared with 194,840 admissions (6.3%) with a primary mental health diagnosis. At

the ZIP-quarter level, the mean count is 93.9 for mental-health-comorbid admissions versus 16.4 for primary mental health admissions.

Vulnerable populations: Following the mechanism that power outages may aggravate conditions that depend on electricity-supported care or daily disease management, we identify five vulnerable groups by principal diagnosis: diabetes (E08–E13; 60,865 admissions, 25.2% with coexisting depression or anxiety), COPD and asthma (J40–J47; 57,682, 33.6%), heart disease (I20–I25 and I50; 72,503, 21.2%), dementia (G30–G31; 3,993, 36.0%), and chronic kidney disease (N18; 541, 21.8%). Within each group we separate admissions with and without coexisting depression or anxiety (F32, F33, F41, or F43) in the secondary diagnosis fields. Dementia is defined using G30–G31 only, because vascular and other dementias coded F01–F03 fall within the mental health definition.

Estimation and results: All outcomes are estimated with the same PPML-HDFE specification as the main results (ZIP code and Year × Quarter fixed effects, total population as the exposure term, standard errors clustered at the ZIP code level), with one modification: we exclude the two Elixhauser comorbidity-index controls, because these indices are constructed from the same secondary diagnosis fields as the outcomes. In this specification, power outages have no detectable effect on mental-health-comorbid admissions (IRR = 0.996, 95% CI: 0.988–1.004), while the effect on primary mental health admissions remains positive (IRR = 1.013, $p < 0.10$) and admissions with no mental health code show a small positive association (IRR = 1.007, $p < 0.05$) (Table S16). The null result for mental-health-comorbid admissions is robust to 24 alternative outage definitions (duration thresholds of 1–8 hours combined with 50th/75th/90th severity percentiles; all IRRs remain near 1.00 [0.996–1.001], none significant) and is unchanged when the Elixhauser indices are retained (IRR = 0.999). In the vulnerable-population analysis (Table S17), point estimates are larger for admissions with coexisting depression or anxiety in three of the five groups, and the estimate for chronic kidney disease with coexisting depression or anxiety is large and statistically significant (IRR = 1.769, $p < 0.05$) but rests on only 118 admissions statewide and should be treated as suggestive.

Supplemental methods

Method S1. ICD-10-CM coding of primary mental health diagnoses, Related to STAR Methods

The International Classification of Diseases, 10th Revision (ICD -10) is a standardized system for classifying diseases, conditions, and health-related issues. It is widely used in healthcare data analysis, including within the Healthcare Cost and Utilization Project (HCUP) databases such as the State Inpatient Databases (SID) and the State Emergency Department Databases (SEDD). The ICD-10 Clinical Modification (ICD-10-CM) is the

U.S. adaptation for morbidity coding, which provides enhanced specificity compared to its predecessor, ICD-9-CM [S31].

The Clinical Classifications Software Refined (CCSR) tool, developed by the Agency for Healthcare Research and Quality (AHRQ), aggregates ICD-10-CM diagnosis codes into clinically meaningful categories to facilitate research on healthcare utilization, costs, quality, and policy evaluation. The use of ICD-10-CM codes in HCUP data ensures that diagnostic information is systematically categorized for robust statistical analysis. By categorizing diagnoses into these structured ICD-10-CM groupings, the study ensures that mental health conditions and related injuries are systematically analyzed across hospital admissions visits.

HCUP SID includes up to 101 diagnosis fields (DX1–DX101). Our primary outcome uses the principal diagnosis (DX1) only, so that mental health is the main recorded reason for hospitalization rather than a secondary or comorbid condition. Under this primary-diagnosis definition, admissions in which a mental health condition appears only in a secondary field (DX2–DX101) are not counted as primary mental health hospitalizations; for example, if DX1 is a cardiovascular condition and anxiety (F41) appears in DX2, the stay is classified as a physical-principal admission. This restriction is intended to capture acute and severe mental health events that drive inpatient utilization and to reduce confounding from admissions where physical conditions are the principal reason for care. In a complementary analysis added in this revision, we separately examine admissions in which mental health appears as a secondary (comorbid) diagnosis across DX2–DX101 (see Note S4 and Tables S16–S17).

To analyze mental health-related hospital encounters, this study utilizes ICD-10-CM codes within HCUP's SID data. The selected codes cover primary mental health conditions:

Table S2 provides a detailed breakdown by mental health condition type, comparing prevalence when conditions are recorded as the primary diagnosis versus any diagnosis position. Primary diagnoses were classified using ICD-10-CM codes beginning with "F" in the first diagnosis field (I10_DX1). Secondary diagnoses were identified from diagnosis fields I10_DX2 through I10_DX101.

Method S2. Construction of percentile-based power outage episode measures, Related to STAR Methods

This section provides a detailed explanation for calculating power outage episodes using data-driven percentile thresholds.

Overview of the Approach

The method we use is based on percentiles of the actual outage distribution and identifies continuous outage episodes that may span multiple time periods. There are mainly four steps:

a. Threshold determination: calculate meaningful outage severity levels from the data itself.

b. Episode identification: group consecutive time periods into continuous outage events

c. Performance measurement: aggregate episodes into quarterly performance metrics

d. Multi-level analysis: generate comparable metrics across different severity levels.

Determining Outage Severity Thresholds

Using fixed thresholds (e.g., “outage affecting more than 5% of customers”) creates several problems. Different geographic areas have vastly different customer bases, seasonal patterns vary across years, and arbitrary cutoffs may miss locally significant events while including trivial ones elsewhere.

Instead, we calculate thresholds directly from the distribution of observed outage rates. We first identify all time periods when actual outages occurred (outage rate > 0) and then calculate key percentiles of this distribution.

Data Source and Study Period

For the power outage data, we obtained the power outage data from year 2014 to 2023 from the State of Maryland. Our study period is from 2018 to 2023.

The original raw data file has the following information

ZIPCODE: Geographic identifier

PROVIDER: Utility company identifier

ARCHIVED: Timestamp of outage record

OUTAGE: number of customers affected

YEAR: calendar year

When the OUTAGE = 0, it represents no current outages.

Estimating Customer Coverage

For the calculation of customers tracked, we follow the approach used by poweroutage.us [S32] (e.g., [S33,S34]). Because many electric utilities do not report the total number of customers that they serve at the local level, this approach defines “Customers Tracked” for each ZIP code- provide pair as the maximum number of customers affected observed historically (2014-2023). This value is used as a proxy for the total customer base in that area.

Estimating historical customer coverage ("Customers Tracked"): In order to normalize outage counts across time and location, I estimate a denominator for each ZIP-PROVIDER pair: for each unique (ZIPCODE, PROVIDER) combination, we calculated the maximum observed outage value across the entire 2014-2023 period. This maximum value was stored as "Customers Tracked", representing the estimated historical capacity or maximum customer base in that ZIP-PROVIDER cell.

Percentile-Based Threshold Calculation

For each observation *i*, the outage rate is:

$$\text{OutageRate}_{\text{i}} = \frac{\text{CustomersAffected}_{\text{i}}}{\text{CustomersTracked}_{\text{zip , provider}}}$$

we then calculate three threshold levels:

- 50th percentile: captures moderate outages (upper half of all outage events)
- 75th percentile: identifies significant outages (upper quarter of all outage events)
- 90th percentile: isolates severe outages (top 10% of all outage events)

This approach ensures that the severity classifications are meaningful relative to the actual patterns in the data, rather than being imposed externally.

This percentile-based approach is consistent with prior literatures that defines outage exposure using the proportion of customers affected and applies percentile thresholds (e.g., 50th or 75th percentile) to distinguish moderate from severe outage events [S27,S29,S35,S36].

Creating Binary Indicators

For each threshold level, I create binary indicators that flag whether each 15-minute observation exceeds that threshold:

$$\text{Flag}_{\text{i, threshold}} = \begin{cases} 1 & \text{if outage rate i} > \text{ threshold} \\ 0 & \text{otherwise} \end{cases}$$

Identifying Continuous Outage Episodes

Raw data consist of 15-minute snapshots. A single outage event might appear as multiple consecutive flagged observations, or there might be brief gaps in reporting during a continuous outage. Simply counting flagged observations would overestimate the number of distinct outage events.

We identify continuous outage episodes by examining the temporal relationship between flagged observations within each ZIP code-provider combination. Two key rules used in episode formation:

- Continuity Rule: Consecutive flagged observations separated by 25 minutes or less are considered part of the same episode. This accounts for minor data collection gaps while preserving episode integrity.
- Duration Calculation: Episode duration is measured as the time span from the first to last observation in the episode, measured in 15-minute intervals:

$$\text{Episode Duration } = \frac{\text{End Time } - \text{ Start Time}}{\text{15 minutes}} + 1$$

Episode Classification

We classify episodes into duration categories that reflect different levels of customer impact.

The analysis generates duration thresholds for 1,2,3,4,5,6,7, and 8 hours, creating variables for each threshold level:

- 1-hour threshold: 4 or more intervals (≥1 hour)
- 2-hour threshold: 8 or more intervals (≥2 hours)
- 3-hour threshold: 12 or more intervals (≥3 hours)
- 4-hour threshold: 16 or more intervals (≥4 hours)
- 5-hour threshold: 20 or more intervals (≥5 hours)
- 6-hour threshold: 24 or more intervals (≥6 hours)
- 7-hour threshold: 28 or more intervals (≥7 hours)
- 8-hour threshold: 32 or more intervals (≥8 hours)

This multi-threshold approach allows for sensitivity analysis across different definition of prolonged outages.

Each original observation is then tagged with information about the episode it belongs to, including episode duration and severity classification. This tagging process helps us aggregate episode-level statistics at the quarterly level.

Constructing Quarterly Outage Performance Metrics

To construct quarterly outage performance metrics, I focus on episodes that begin within each quarter, creating the variable "new_episodes_p75" for episodes identified using the 75th percentile threshold. This "new episodes" approach addresses a critical measurement challenge: outage episodes can span across time boundaries, potentially starting in one quarter and ending in the next.

Calculation of New Episodes: For each ZIP code and quarter, new_episodes_p75 counts the number of distinct outage episodes whose first observation falls within that quarter's date range. Specifically, for an episode spanning multiple time periods, I identify the earliest timestamp in the episode and assign the entire episode to whichever quarter contains that start date.

This methodology ensures that episodes are assigned to the quarter corresponding to their start time, so that events spanning multiple quarters are counted only once rather than being double-counted. For example, if an outage episode begins on March 30th (Q1) and continues until April 2nd (Q2), this episode contributes 1 to the new_episodes_p75 count for Q1 and 0 to the Q2 count, even though both quarters experienced the outage.

This approach provides a more accurate measure of outage initiation patterns, which is particularly relevant for analysing the effectiveness of preventive maintenance or system improvements. The 75th percentile threshold (p75) means that new_episodes_p75 specifically counts episodes where the outage rate exceeded the threshold that captures the most significant 25% of all observed outage events in the dataset. This focuses the analysis on significant outages while filtering out minor fluctuations that may not meaningfully impact customer experience.

Primary Exposure Variable

Our primary exposure variable is the count of new severe outage episodes per ZIP-quarter (new_1hr_episodes_p75), which represents distinct outage events lasting one hour or longer that exceed the 75th percentile severity threshold within each ZIP-quarter period. Given the count nature of this variable and the presence of substantial zero-inflation (many ZIP-quarters experience no severe outages), I apply a log transformation (the constant 1 is added to accommodate zero values):

$$\ln \text{ outage}_{it} = \ln(\text{ new\_1hr\_episodes\_p } 75_{it} + 1)$$

This transformation serves several purposes: (1) it handles the numerous zero observations by mapping them to zero on the log scale, (2) it normalizes the highly right-skewed distribution typical of count data, and (3) it allows for more intuitive interpretation of coefficients as approximate percentage changes in mental health outcomes associated with changes in outage frequency.

Method S3. Weather data collection and processing, Related to STAR Methods

Data Collection Process

The weather data for this analysis was obtained from the National Oceanic and Atmospheric Administration (NOAA) through the Climate Data Online (CDO) web API. The data extraction process utilized a python-based program to retrieve historical weather observations from the Global Historical Climatology Network – Daily (GHCN-Daily) dataset for all available weather stations in Maryland. To manage API rate limitations imposed by NOAA’s service, the program used unique API tokens to retrieve the data.

The resulting dataset was structured in a wide format optimized for spatial analysis in ArcGIS Pro, where each row represents a unique combination of station and date, with weather variables organized as separate columns. Each weather station's data records contain the station's geographic coordinates (latitude and longitude) and daily weather observations.

Spatial Data Processing and Geographic Attribution

Following data collection, weather stations were assigned to ZIP code areas for geographic aggregation. The station coordinates (latitude and longitude) from the NOAA data were converted to point features in ArcGIS Pro using the "XY to Point" tool. These points were then spatially joined with ZIP Code Tabulation Area (ZCTA) boundaries from the U.S. Census Bureau's TIGER/Line Shapefiles, obtained from the official Census repository. This spatial join process determined which ZIP code area contained each weather station based on its geographic location. The resulting dataset linked each weather station's daily observations to its corresponding ZIP code, enabling aggregation of weather data by postal regions for subsequent analysis with power outage data at the same geographic scale.

Following the documentation of the Global Historical Climatology Network – Daily dataset maintained by NOAA, these variables are defined as follows:
The precipitation variable (PRCP) represents the daily total precipitation amount, encompassing all forms of liquid and liquid-equivalent frozen precipitation. This measurement captures rainfall, drizzle, and the water equivalent of snow, sleet, and hail, reported in tenths of millimeters in the raw data.

The snowfall variable (SNOW) measures the daily snowfall amount, representing the vertical depth of new snow accumulated during the 24-hour observation period, similarly reported in millimeters with conversion options available.

Temperature extremes are captured through the maximum temperature variable (TMAX), which records the highest air temperature observed during each 24-hour period. This variable is essential for identifying heat stress conditions and temperature-related infrastructure impacts, with values reported in tenths of degrees Celsius in the original format.
Wind conditions are represented through the fastest 2-minute wind speed variable (WSF2), which records the highest average wind speed measured over any consecutive 2-minute interval during the observation period. This variable captures sustained high winds rather than brief gusts, making it particularly relevant for assessing wind-related infrastructure stress and power outage risk. These measurements are reported in tenths of meters per second.

The daily weather observations were aggregated to quarterly frequency to align with the resolution of HCUP SID discharge quarterly data. The daily weather data from each weather station was converted to quarterly (3-month) averages to match the time period used in power outage reports. For each ZIP code area and quarter, I calculated the average maximum temperature by taking the mean of all daily high temperatures during that quarter. Similarly, I calculated average precipitation and wind speed for each quarter. I also calculated total precipitation and total snowfall by adding up all the daily amounts within each quarter.

To identify what counts as "extreme" weather, I analyzed all weather data from 2018-2023 and found the threshold values that separate normal from unusual conditions. I used three levels of severity:

- **95th percentile**: The most extreme 5% of weather (temperatures above [p95 value is 90.77] °F for heat extremes)
- **90th percentile**: The most extreme 10% of weather (temperatures above [p90 value is 88.24]°F)
- **75th percentile**: The most extreme 25% of weather (temperatures above [75 value is 81.87]°F)

Note: Fore precipitation, snowfall, and wind speed, the percentile thresholds were calculated only among days with non-zero values (i.e., days with actual precipitation, snowfall, or mesurable wind), following standard meteorological practice to avoid skewing thresholds with zero-value observations.
These thresholds mean that historically, only 5% of days had maximum temperatures exceeding 90.77°F, 10% exceeded 88.24°F, and 25% exceeded 81.87°F. For cold extremes, I used the opposite approach - the 5th percentile (p5 value is 38.75°F) represents the coldest 5% of days, the 10th percentile (p10 value is 43.45°F) the coldest 10%, and the 25th percentile (p25 value is 52.22°F) the coldest 25%.

For example, if the maximum temperature on a given day is 92°F, that day would be classified as an "extreme heat day" at the 95th percentile level (since 92°F exceeds the 90.77°F threshold). We then counted the total number of such extreme weather days occurring within each quarter for each ZIP code area, creating variables that measure the frequency of extreme weather events at different severity levels.

Counting Extreme Weather Days

For each day in our dataset, we checked whether the weather exceeded our threshold values. If the daily maximum temperature was above the 90th percentile threshold, we marked that as an extreme heat day. We then counted how many extreme weather days occurred in each quarter for each ZIP code. This gave us variables at three severity levels:

95th Percentile (Most Extreme 5%):

- extreme_heat_days_p95: Number of days with temperatures above the 95th percentile (>90.77°F)
- extreme_cold_days_p95: Number of days with temperatures below the 5th percentile (<38.75°F)
- extreme_precip_days_p95: Number of days with precipitation above the 95th percentile
- extreme_wind_days_p95: Number of days with wind speed above the 95th percentile
- extreme_snow_days_p95: Number of days with snowfall above the 95th percentile
-

90th Percentile (Most Extreme 10%):

- extreme_heat_days_p90: Number of days with temperatures above the 90th percentile (>88.24°F)
- extreme_cold_days_p90: Number of days with temperatures below the 10th percentile (<43.45°F)
- extreme_precip_days_p90: Number of days with precipitation above the 90th percentile
- extreme_wind_days_p90: Number of days with wind speed above the 90th percentile
- extreme_snow_days_p90: Number of days with snowfall above the 90th percentile
-

75th Percentile (Most Extreme 25%):

- unusual_heat_days_p75: Number of days with temperatures above the 75th percentile (>81.87°F)
- unusual_cold_days_p75: Number of days with temperatures below the 25th percentile (<52.22°F)
- unusual_precip_days_p75: Number of days with precipitation above the 75th percentile
- unusual_wind_days_p75: Number of days with wind speed above the 75th percentile
- unusual_snow_days_p75: Number of days with snowfall above the 75th percentile

Aggregate Extreme Weather Variables

To create a comprehensive measure of overall extreme weather exposure, we calculated total extreme weather days by summing across all weather types within each percentile threshold. For each ZIP code-quarter observation:

- extreme_weather_total_days_p95 = extreme_heat_days_p95 + extreme_cold_days_p95 + extreme_precip_days_p95 + extreme_wind_days_p95 + extreme_snow_days_p95
- extreme_weather_total_days_p90 = extreme_heat_days_p90 + extreme_cold_days_p90 + extreme_precip_days_p90 + extreme_wind_days_p90 + extreme_snow_days_p90
- unusual_weather_total_days_p75 = unusual_heat_days_p75 + unusual_cold_days_p75 + unusual_precip_days_p75 + unusual_wind_days_p75 + unusual_snow_days_p75
-

These composite variables capture the cumulative burden of multiple extreme weather events, recognizing that quarters with several types of extreme conditions may pose greater risks than those with only one type.

Data Source: NOAA. (2025). *Global Historical Climatology Network – Daily Documentation*. National Centers for Environmental Information. Retrieved from http://www1.ncdc.noaa.gov/pub/data/ghcn/daily/

Method S4. Geographic disaggregation of healthcare resources from county to ZIP code level, Related to STAR Methods

This study employs a geographic disaggregation methodology to transform county-level healthcare resource data into ZIP code-level estimates, enabling more granular spatial analysis of healthcare accessibility. The transformation process utilizes data from the Area Health Resources Files (AHRF), which provides comprehensive healthcare workforce and facility information aggregated at the county level, and combines it with the HUD-USPS ZIP Code Crosswalk files to achieve finer geographic resolution at the ZIPCODE level.

The AHRF dataset (https://data.hrsa.gov/topics/health-workforce/nchwa/ahrf), maintained by the Health Resources and Services Administration, integrates information from over 50 data sources encompassing healthcare professions, health facilities, population characteristics, economics, hospital utilization, and expenditures at county, state, and national levels. To account for regional variations in healthcare infrastructure that may influence both baseline mental health hospitalization patterns and the response to power outages, we incorporated healthcare resource measures from the Area Health Resources File (AHRF). The AHRF, maintained by the Health Resources and Services Administration (HRSA), provides comprehensive county-level data on health system capacity by compiling information from multiple federal sources including the Census Bureau and Centers for Disease Control and Prevention.

Given that our analysis for Maryland power outage and inpatient data are at the ZIP code level, we implement a proportional allocation methodology using the HUD-USPS ZIP Code Crosswalk files developed by the U.S. Department of Housing and Urban Development's Office of Policy Development and Research (https://www.huduser.gov/portal/datasets/usps_crosswalk.html). These crosswalk files provide a unique linkage between USPS ZIP codes and Census geographies, derived from quarterly USPS Vacancy Data that captures the spatial distribution of both business and residential addresses. Importantly, these files include a residential ratio (RES_RATIO) that indicates the proportion of residential addresses in each ZIP code relative to its parent county, enabling a more nuanced allocation approach than simple area-based or population-based methods.

The transformation process begins by expanding the annual county-level healthcare resource data into quarterly observations to align with the temporal structure of the HUD-USPS crosswalk files, which are updated quarterly to reflect changes in ZIP code configurations. The county-level data is then merged with the crosswalk files using county, year, and quarter variables, creating multiple records for each original county observation corresponding to all ZIP codes that overlap with that county.

The core of the disaggregation methodology involves proportionally allocating county-level healthcare resources to ZIP codes based on the residential address ratio. For each healthcare resource variable, the county-level value is multiplied by the RES_RATIO to estimate the portion of resources attributable to each ZIP code.

We extracted three key indicators of healthcare supply: the number of psychiatrists, total hospital bed capacity combining both short-term general hospital beds and long-term hospital beds, and the number of general practice physicians providing patient care. Since the AHRF provides data at the county level while the Maryland SID analysis requires ZIP code-level granularity to match the power outage and hospitalization data, we implemented a geographic disaggregation using the HUD-USPS ZIP Code Crosswalk files. The core of this methodology involves proportionally allocating county-level healthcare resources to ZIP codes based on the residential address ratio (RES_RATIO). For each healthcare resource variable, the county-level value is multiplied by the RES_RATIO to estimate the portion of resources attributable to each ZIP code:

$$\text{ZIP-level Resource} = \text{County-level Resource} \times \text{RES RATIO}$$

Because some ZIP codes span multiple counties, a single ZIP code may receive partial allocations from two or more counties. We therefore aggregate these partial allocations by summing across all contributing counties, ensuring that each ZIP code's total healthcare resources reflect contributions from all overlapping counties while preserving the total resource distribution. The resulting ZIP code-level estimates were

then converted to population-adjusted rates per 100,000 residents using corresponding population estimates to facilitate comparisons across geographic areas of varying sizes. Healthcare infrastructure may influence both baseline mental health hospitalization patterns and the community's capacity to respond to power outages. Areas with limited mental health resources may experience disproportionate surges in emergency department visits and hospitalizations when routine outpatient services become unavailable during outages.

We consider three indicators of healthcare supply. Psychiatrist density captures the availability of specialized mental health services, which may influence both routine admission patterns and hospitals' capacity to manage mental health crises during power outages. Hospital bed availability reflects overall inpatient capacity, where higher bed density may lower admission thresholds and enable greater utilization during emergencies. General practitioner availability represents primary care accessibility, as these providers often serve as the first point of contact for mental health concerns and may provide crucial continuity of care when specialized services are disrupted.

However, due to high multicollinearity among these three healthcare resource indicators—reflecting the tendency for areas with more psychiatrists to also have greater hospital capacity and primary care availability—we included only one resource variable in each model specification to avoid variance inflation issues while still controlling for healthcare infrastructure effects.

Method S5. Elixhauser comorbidity index, Related to STAR Methods

To isolate the relationship between power outages and mental health-related emergency department (ED) visits and mental health hospitalization rate, we incorporated the Elixhauser Comorbidity Index (ECI). The ECI data was obtained from the Healthcare Cost and Utilization Project (HCUP-US), specifically utilizing the Elixhauser Comorbidity Software Refined for ICD-10-CM. The ECI covers 38 comorbidities (in the refined ICD-10-CM version), including non-mental health conditions (e.g., CM_CHF for congestive heart failure, CM_DIABETES_C for complicated diabetes) that may influence ED visits and hospitalization rate during power outages. This allows us to control for confounding physical health conditions that could mediate outage effects (e.g., diabetes patients needing insulin refrigeration or cardiac patients relying on medical devices), which is critical for isolating mental health effects.

To adjust for patient-level health status, we include the CMR_Index_Readmission (Comorbidity Software Refined readmission index), a weighted Elixhauser comorbidity index developed by HCUP based on ICD-10 diagnosis codes. This index aggregates over the 38 comorbid conditions (e.g., heart failure, diabetes, chronic lung disease), each assigned a coefficient derived from logistic models predicting 30-day readmission risk. The resulting continuous variable reflects the overall burden of

comorbidities, with higher values indicating worse baseline health and greater hospitalization risk. Including this index allows us to control for heterogeneity in underlying health conditions across patients. The CMR_Index_Readmission controls for overall health vulnerability, ensuring that observed effects are not simply driven by areas with sicker populations having both more outages and more ED visits.

In addition, to assess the prevalence of mental health-related comorbidities within each geographic and temporal unit, we utilized the Elixhauser Comorbidity Software provided by the HCUP. The Maryland State Inpatient Database (SID) Core files were processed through the official SAS program to generate binary indicators for each admission (per discharge), identifying the presence of specific comorbid conditions based on ICD diagnosis codes. Among the generated indices, we focused on three mental health-related comorbidities: psychoses (CM_PSYCHOSES), alcohol-related disorders (CM_ALCOHOL), and drug abuse disorders (CM_DRUG_ABUSE).

Following the generation of patient-level comorbidity indicators, we aggregated the data to the ZIP code-year-quarter level. For each comorbidity variable, we calculated the mean value across all admissions within each geographic-temporal unit, which yields the proportion of admissions presenting with each specific comorbidity. These proportions were subsequently multiplied by 100 to express them as percentages. The variables pct_psychoses, pct_alcohol, and pct_drug_abuse represent the percentage of total admissions within each ZIP code-year_quarter combination that presented with psychoses, alcohol-related disorders, and drug abuse disorders, respectively.

Method S6. Construction of instrumental variables: power grid infrastructure density, Related to STAR Methods

To address potential endogeneity in local power outage exposure, this study employs infrastructure-based instrumental variables capturing exogenous variation in the physical structure of the electric power network. Specifically, we construct four ZIP-code-level measures: transformer density, distribution line density, substation density, and high-voltage transmission line density.

All infrastructure variables are derived using Geographic Information System (GIS) spatial overlays between power grid infrastructure shapefiles and ZIP Code Tabulation Area (ZCTA) boundaries.

Spatial Aggregation Procedure

For each ZIP code *i*, we compute infrastructure density measures using the following steps:

1. Spatial clipping – National electric grid infrastructure shapefiles are spatially intersected with ZIP code boundaries. Each infrastructure element (point or line) is assigned to a ZIP code based on geographic location.

2. Infrastructure quantification within ZIP codes

- Line-based infrastructure (transmission lines and distribution lines): the total lengths of lines (in kilometers) within each ZIP code is calculated.
- Point-based infrastructure (substations and transformer): the number of facilities located within each ZIP code is counted.

3. Normalization by land area

   To ensure comparability across ZIP codes of different sizes, each infrastructure measure is converted into a density measure by dividing by the ZIP code's land area (in square kilometers).

Instrumental Variable Definitions

(1) Substation Density

$$\text{Substation Density}_{\,i} = \frac{\text{Number of substations in ZIP i}}{\text{Land area of ZIP i}(\text{km}^2)}$$

Substations are facilities that switch, transform, or regulate electric power, typically operating at voltages ≥ 69 kV, and serve as key nodes in the high-voltage transmission network.

(2) High-Voltage Transmission Line Density

$$\text{Transmission Line Density}_{\,i} = \frac{\text{Total length of transmission lines in ZIP i}(\text{km})}{\text{Land area of ZIP i}(\text{km}^2)}$$

Transmission lines are high-voltage (69–765 kV) lines designed to transport large quantities of electricity over long distances between substations and major load centers.

(3) Transformer Density

$$\text{Transformer Density}_{\,i} = \frac{\text{Number of distribution transformers in ZIP i}}{\text{Land area of ZIP i}(\text{km}^2)}$$

Distribution transformers step down voltage from distribution feeders to levels usable by end consumers and represent the local last-mile delivery infrastructure of the electric grid.

(4) Distribution Line Density

$$\text{Distribution Line Density}_{\,i} = \frac{\text{Total length of distribution lines in ZIP i}(\text{km})}{\text{Land area of ZIP i}(\text{km}^2)}$$

Distribution lines are medium- and low-voltage lines (typically < 35 kV) that deliver electricity from substations to transformers and ultimately to households and businesses through local distribution networks.

Interpretation of Infrastructure-Based Instruments

These instruments capture pre-existing, slow-moving physical grid topology rather than short-term demand fluctuations or local health conditions. Importantly:

- Transmission infrastructure reflects system-level grid structure, which influences the likelihood of large-scale and long-duration outages under extreme stress.

- Distribution infrastructure reflects local network density, which is more closely associated with frequent, shorter-duration service disruptions due to localized faults or overloads.

  Because the physical layout of grid infrastructure is determined by long-run engineering and regulatory planning rather than contemporaneous mental health trends, these measures provide plausibly exogenous variation in outage exposure.

Data Sources

Substations data source:
https://www.arcgis.com/home/item.html?id=6511edc77ec3430493cce610ab48f0c1
Transmission Lines Source:
https://www.arcgis.com/apps/mapviewer/index.html?layers=d4090758322c4d32a4cd002ffaa0aa12
Transformer and distribution lines:
Distribution Infrastructure and Transformers are compiled from regional utility GIS datasets.

Figure S1. IV Second Stage Results - Alternative Outage Definitions, Related to Table 2

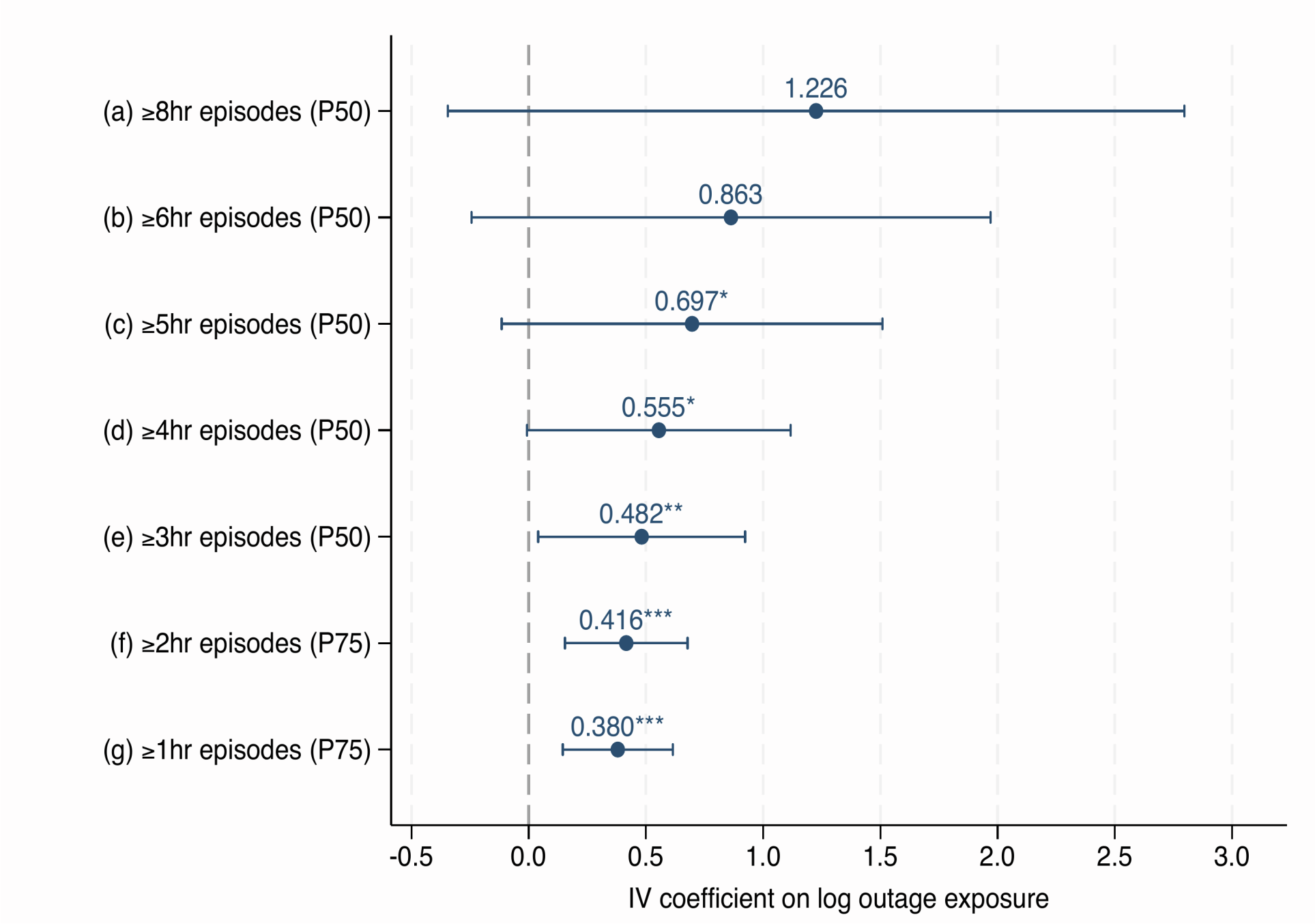

Notes: Second-stage coefficients from IV Poisson GMM with 95% confidence intervals. Standard errors clustered at ZIP code level. IV includes transformer density, transmission line density, and substation density. First-stage F-statistics: 19.76 (≥1hr P75), 17.12 (≥2hr P75), 16.59 (≥3hr P50), 14.23 (≥4hr P50), 11.19 (≥5hr P50), 8.95 (≥6hr P50), 7.89 (≥8hr P50). All Hansen J overidentification tests pass (p-values range from 0.399 to 0.948). All outage measures are log-transformed as ln(measure + 1). Outage definitions: "≥Xhr episodes (P50/P75)" indicates the number of outage episodes lasting at least X hours with severity above the 50th or 75th percentile within each ZIP code-quarter. All models include year fixed effects and controls for extreme heat days, psychoses prevalence, alcohol use disorder prevalence, poverty rate, percent male, median income, psychiatrist supply, comorbidity indices, racial composition, and insurance coverage. * $p < 0.10$, ** $p < 0.05$, *** $p < 0.01$

Table S1. Construction of primary mental health diagnosis indicators using ICD-10-CM principal diagnosis codes (I10_DX1), Related to STAR Methods

**Condition**	**ICD-10 Code Range (I10_DX1)**	**Definitions**
Primary_Anxiety_PTSD	F41, F43	Disorders Anxiety disorders (F41) and reactions to severe stress, including PTSD and adjustment disorders (F43).
Primary_Depression	F32, F33	Depressive episodes (F32) and recurrent depressive disorder (F33).
Primary_Substance_Use	F10–F19	Mental and behavioral disorders due to psychoactive substance use, including alcohol, opioids, cannabis, sedatives, stimulants, and other substances.
Primary_Schizophrenia_Psychotic	F20, F22–F25, F28, F29	Schizophrenia and other non-mood psychotic disorders.
Primary_Bipolar_Disorder	F30, F31	Manic episodes (F30) and bipolar affective disorder (F31).
Primary_Developmental_Behavioral	F70–F79, F80–F89, F90–F95	Developmental and behavioral disorders, including intellectual disabilities and neurodevelopmental disorders.
Primary_Personality_Impulse	F60–F63, F68	Personality disorders and impulse-control disorders, including enduring maladaptive personality traits and difficulties in regulating behavior.
Primary_Dissociative_Somatoform	F44, F45, F48	Dissociative and somatoform disorders, characterized by disruptions in identity or physical symptoms without clear medical causes.
Primary_Other_Mood	F34, F39	Other mood (affective) disorders, including persistent or unspecified mood disturbances not classified as major depression or bipolar disorder.
Primary_OCD	F42	Obsessive-compulsive disorder, characterized by recurrent intrusive thoughts and repetitive behaviors.

Primary_Eating_Disorders	F50	Eating disorders involving abnormal eating behaviors that adversely affect physical and mental health.
Primary_Other_Mental_Health	Other diagnoses starting with 'F' that are not included in the above categories.	All remaining ICD-10 mental health diagnoses (F00–F99) not captured by the above categories, including organic mental disorders, sleep disorders, and other unspecified conditions.

Table S2. Distribution of Mental Health (MH) Diagnoses Among Inpatient cases, Maryland 2018-2023, Related to STAR Methods

Category	N	% of Total Cases
Total Inpatient Cases	3,075,969	100.00%
Mental Health Diagnosis Position		
Any MH diagnosis (primary or secondary)	1,301,068	42.30%
Primary MH diagnosis only	194,840	6.33%
Secondary MH diagnosis only	1,106,228	35.96%
No MH diagnosis	1,774,901	57.70%

Note: Primary diagnosis refers to the principal diagnosis (I10_DX1) recorded for the hospitalization. Secondary diagnosis include all other diagnosis positions (I10_DX2 through I10_DX101). MH = Mental Health

Table S3. Prevalence of Mental Health Conditions by Diagnosis Type, Maryland 2018-2023, Related to STAR Methods

Mental Health Conditions	ICD-10 Codes	Primary Diagnosis	Any Diagnosis Position
		N (%)	N (%)
Mood Disorders			
Depression	F32-F33	48,566 (1.58%)	582,793 (18.95%)
Bipolar Disorder	F30-F31	38,170 (1.24%)	-
Other Mood Disorders	F34, F39	3,418 (0.11%)	-
Anxiety & Stress Related			
Anxiety & PTSD	F41, F43	7,094 (0.23%)	525,077 (17.07%)
Stress-Related	F43	-	80,728 (2.62%)

Disorders			
Substance Use Disorders	F10-F19	45,034 (1.46%)	603,189 (19.61%)
Psychotic Disorders			
Schizophrenia & Psychotic	F20-F29	41,110 (1.34%)	89,154 (2.90%)
Other Mental Health Conditions			
Developmental & Behavioral	F70-F95	1,519 (0.05%)	-
Dissociative & Somatoform	F44-F45, F48	1,222 (0.04%)	-
Personality & Impulse	F60-F63, F68	883 (0.03%)	-
OCD	F42	189 (0.01%)	-
Eating Disorders	F50	484 (0.02%)	-
Other Mental Health	Other F codes	7,151 (0.23%)	-
Total	All F codes	194,840 (6.33%)	1,301,068 (42.30%)

Notes: Percentages calculated as proportion of total inpatient cases (N = 3,075,969). "Any Position" percentages are not mutually exclusive as patients may have multiple diagnoses. "—" indicates not separately calculated.

* "Any Position" for Depression includes all F30-F39 codes (mood disorders including bipolar).

** "Any Position" for Anxiety includes F40-F48 codes.

Table S4. Extreme Weather Thresholds, Related to STAR Methods

Variable	75th Percentile	90th Percentile	95th Percentile
Maximum Temperature (°F) - Heat	81.87	88.24	90.77
Maximum Temperature (°F) - Cold	52.22 (25th)	43.45 (10th)	38.75 (5th)
Precipitation (inches)	0.27	0.68	1.03
Wind Speed (m/s)	20.36	24.87	28.03
Snowfall (mm)	0.80	2.22	3.67

Table S5. Summary Statistics - Full Variable List (ZIP-Quarter Level), Related to STAR Methods

Variable Names	Definitions	N	Mean	SD
mh_primary_cases	Count of inpatient admissions with a primary (principal) mental health diagnosis per ZIP code-quarter	11737	16.45	29.07
new_1hr_episodes_p75	Number of power outage episodes lasting ≥1 hour at 75th percentile intensity threshold	9336	5.77	5.32
ln_outage_1hr_p75	Natural log of power outage episodes lasting ≥1 hour at 75th percentile threshold, ln(new_1hr_episodes_p75 + 1)	9336	1.62	0.79
extreme_heat_days_p90	Number of days with maximum temperature above 90th percentile (>88.24°F)	9091	8.48	12.78
avg_cmr_clean	Mean Elixhauser Comorbidity Index for readmission risk	11701	7.47	3.10
avg_cmr_mortality_clean	Mean Elixhauser Comorbidity Index for mortality risk	10938	5.34	4.06
pct_psychoses	Percentage of admissions flagged with psychoses comorbidity (Elixhauser CM_PSYCHOSES, %)	11737	3.99	6.99
pct_alcohol	Percentage of admissions flagged with alcohol abuse (Elixhauser CM_ALCOHOL, %)	11737	4.96	7.04
pct_drug_abuse	Percentage of admissions flagged with drug abuse (Elixhauser CM_DRUG_ABUSE, %)	11737	4.71	7.80
pct_diabetes_cx	Percentage of admissions flagged with diabetes with chronic complications (Elixhauser CM_DIAB_CX, %)	11737	16.74	12.95
pct_hypertension_cx	Percentage of admissions flagged with complicated hypertension (Elixhauser CM_HTN_CX, %)	11737	20.92	14.50
pct_copd	Percentage of admissions flagged with chronic pulmonary disease (Elixhauser CM_LUNG_CHRONIC, %)	11737	19.61	13.73
pct_renal_disease	Percentage of admissions flagged with moderate renal failure (Elixhauser CM_RENLFL_MOD, %)	11737	10.21	10.38

median_income_1k	Median household income in ZIP code (ACS, in $1,000s)	10880	97.62	40.39
poverty_rate	Percent of individuals below the U.S. Census poverty threshold (ACS, %)	11489	8.89	10.07
total_population	Total population in ZIP code (ACS 5-year estimates)	11737	13417	16078
mean_age	Mean age of patients among inpatient admissions (years)	11737	51.98	11.04
pct_male	Percentage of male patients among inpatient admissions (%)	11737	44.98	16.88
black_pct	Share of Black population in ZIP code (ACS, proportion 0–1)	11589	0.18	0.22
asian_pct	Share of Asian population in ZIP code (ACS, proportion 0–1)	11589	0.04	0.06
pct_medicaid	Percentage of hospital admissions covered by Medicaid (%)	11737	20.96	16.74
pct_uninsured	Percentage of self-pay and no-charge inpatient admissions (%)	11737	1.28	3.45
psych_per_100k	Number of psychiatrists per 100,000 population (AHRF; winsorized at 0–99th percentile by year)	11312	10.90	13.91

Notes: Sample includes Maryland data from 2018-2023. Patient-level variables (pct_male, pct_medicaid, mean_age, Elixhauser comorbidities) are calculated as means across all admissions within each ZIP code-quarter. ZIP code-level variables (population, poverty_rate, race percentages) are obtained from ACS 5-year estimates. Healthcare resource variables are from AHRF. See Appendices D-E for detailed variable construction. The number of observations differs across variables because of variable-specific missingness, and the descriptive statistics are based on all available data; the regression estimation sample (Table S9) is smaller (N = 8,152) after merging all covariates and dropping singleton fixed-effect groups. Psychiatrist supply is winsorized at the 0–99th percentile by year, consistent with the regression specifications.

Table S6. Correlation Matrix, Related to STAR Methods

	mh_primary_cases	ln_outage_1hr_p75	extreme_heat_days_p90	poverty_rate	median_income_1k	avg_cmr ean
mh_primary_cases	1					
ln_outage_1hr_p75	0.329***	1				
extreme_heat_days_p90	0.0418***	0.211***	1			
poverty_rate	0.356***	0.107***	0.0109	1		
median_income_1k	-0.210***	0.00450	-0.0345**	-0.573***	1	
avg_cmr_clean	0.137***	0.0592***	-0.0462***	0.194***	-0.161***	1
pct_medicaid	0.300***	0.0611***	0.0505***	0.458***	-0.472***	-0.016
pct_male	-0.0803***	-0.0179***	-0.00408	-0.0334**	0.0240*	0.237**

psych_per_100k	0.220***	0.0431***	0.0285**	0.0518***	0.160***	0.0613*

* $p < 0.05$, ** $p < 0.01$, *** $p < 0.001$

Table S7. Correlation Matrix for instrumental variables, Related to STAR Methods

	ln_outage_1hr_p75	dens_substation	dens_transmission_line	dens_transformer
ln_outage_1hr_p75	1.0000			
dens_substation	0.210***	1.0000		
dens_transmission_line	0.188***	0.578***	1.0000	
dens_transformer	-0.118***	-0.114***	-0.140***	1.0000

* $p < 0.05$, ** $p < 0.01$, *** $p < 0.001$

Table S8. VIF Test, Related to STAR Methods

**Variable**	**VIF**
ln_outage_1hr_p75	1.11
extreme_heat_days_p90	1.06
avg_cmr_clean	2.10
avg_cmr_mortality_clean	1.93
poverty_rate	1.79
pct_male	1.11
median_income_1k	2.15
black_pct	1.29
asian_pct	1.48
pct_medicaid	1.64
pct_uninsured	1.07
psych_per_100k	1.22
**Mean VIF**	**1.49**

Table S9. PPML Results - Mental Health Hospitalizations and Power Outages, Related to Figure 1

Model<br>Dependent Variables	(1)<br>Basic	(2)<br>Weather	(3)<br>Main	(4)<br>Alternative weather	(5)<br>All control variables
ln_outage_1hr_p75	1.017**	1.017**	1.015**	1.016**	1.013*
	(0.008)	(0.008)	(0.008)	(0.008)	(0.007)
extreme_heat_days_p90		1.003***	1.002**		
		(0.001)	(0.001)		
avg_cmr_clean	1.049***	1.049***	1.050***	1.050***	
	(0.008)	(0.008)	(0.008)	(0.008)	
avg_cmr_mortality_clean	0.939***	0.939***	0.943***	0.942***	0.972***
	(0.005)	(0.005)	(0.005)	(0.005)	(0.004)
poverty_rate	1.006*	1.006*	1.008***	1.008***	1.008***
	(0.003)	(0.003)	(0.003)	(0.003)	(0.003)
pct_male			1.006***	1.006***	1.005***
			(0.001)	(0.001)	(0.001)
median_income_1k	1.001	1.001	1.002*	1.002*	1.001
	(0.001)	(0.001)	(0.001)	(0.001)	(0.001)
black_pct			0.970	0.983	
			(0.293)	(0.297)	
asian_pct			0.331**	0.340**	
			(0.157)	(0.161)	
pct_medicaid			1.010***	1.010***	1.006***
			(0.001)	(0.001)	(0.001)
pct_uninsured			1.009***	1.009***	1.005*
			(0.003)	(0.003)	(0.003)
psych_100k			1.025***	1.025***	1.028***
			(0.005)	(0.005)	(0.005)
extreme_precip_days_p90		1.002			
		(0.002)			
extreme_cold_days_p90		0.998			
		(0.001)			
unusual_heat_days_p75				1.001	
				(0.001)	
extreme_heat_days_p95					1.003***
					(0.001)
pct_psychoses					1.027***
					(0.002)
pct_alcohol					1.036***
					(0.003)
pct_diabetes_cx					0.996***
					(0.001)

pct_hypertension_cx					0.997**
					(0.001)
pct_copd					0.999
					(0.001)
Observations	8,276	8,152	8,152	8,152	8,152
ZIP FE	YES	YES	YES	YES	YES
Year*Quarter FE	YES	YES	YES	YES	YES

Notes: Robust standard errors in parentheses, clustered at ZIP code level. *** p<0.01, ** p<0.05, * p<0.1. Fixed effects: ZIP code and Year×Quarter. Exposure: total population.

Table S10. Robustness - Different Outage Thresholds, Related to Figure 2

	(1)	(2)	(3)
	1hr P50	1hr P90	2hr P50
Variable Names			
ln_outage_1hr_p50	1.023***		
	(0.009)		
ln_outage_1hr_p90		1.016***	
		(0.006)	
ln_outage_2hr_p50			1.018**
			(0.008)
extreme_heat_days_p90	1.002**	1.002**	1.002**
	(0.001)	(0.001)	(0.001)
avg_cmr_clean	1.050***	1.050***	1.049***
	(0.008)	(0.008)	(0.008)
avg_cmr_mortality_clean	0.943***	0.943***	0.943***
	(0.005)	(0.005)	(0.005)
poverty_rate	1.008***	1.008***	1.008***
	(0.003)	(0.003)	(0.003)
pct_male	1.006***	1.006***	1.006***
	(0.001)	(0.001)	(0.001)
median_income_1k	1.002*	1.002*	1.002*
	(0.001)	(0.001)	(0.001)
black_pct	0.988	0.959	0.981
	(0.296)	(0.289)	(0.294)
asian_pct	0.323**	0.331**	0.323**
	(0.154)	(0.157)	(0.154)
pct_medicaid	1.010***	1.010***	1.010***
	(0.001)	(0.001)	(0.001)
pct_uninsured	1.009***	1.009***	1.009***
	(0.003)	(0.003)	(0.003)
psych_100k	1.024***	1.025***	1.024***

	(0.006)	(0.005)	(0.006)
Observations	8,152	8,152	8,152
ZIP FE	YES	YES	YES
Year*Quarter	YES	YES	YES
Exposure	Population	Population	Population

Note: * p < 0.10, ** p < 0.05, *** p < 0.01. Robust standard errors in parentheses, clustered at the ZIP code level. All models include ZIP code and Year × Quarter fixed effects.

Table S11. Outage Episodes by Duration Threshold (Log-transformed), Related to STAR Methods

**Variable Names**	**Mean**	**SD**	**Min**	**p50**	**Max**
ln_outage_1hr_p50	2.20	0.91	0.00	2.20	4.76
ln_outage_2hr_p50	1.74	0.93	0.00	1.79	4.57
ln_outage_3hr_p50	1.40	0.92	0.00	1.39	4.29
ln_outage_4hr_p50	1.16	0.89	0.00	1.10	4.09
ln_outage_5hr_p50	0.97	0.86	0.00	0.69	3.89
ln_outage_6hr_p50	0.81	0.82	0.00	0.69	3.76
ln_outage_7hr_p50	0.69	0.78	0.00	0.69	3.64
ln_outage_8hr_p50	0.59	0.74	0.00	0.00	3.58
N	9336				

Table S12. PPML results at the city level (aggregation robustness check), Related to Figure 3

	(1)
Dependent Variables	City*Year FE + Year*Quarter FE
ln_outage_1hr_p75	1.031**
	(0.012)
extreme_heat_days_p90	0.999
	(0.001)
avg_cmr_clean	1.110***
	(0.018)
avg_cmr_mortality_clean	0.922***
	(0.010)
poverty_rate	1.000
	(0.003)
pct_male	1.008***
	(0.002)
median_income_1k	0.995***
	(0.001)
black_pct	1.225**
	(0.115)

asian_pct	0.734
	(0.461)
pct_medicaid	1.006*
	(0.003)
pct_uninsured	1.022**
	(0.011)
psych_100k	1.006
	(0.003)
Observations	7,681
City*Year FE	YES
Year*Quarter FE	YES
Exposure	Population

Note: The dependent variable is the number of hospitalizations with a primary mental health diagnosis. Models are estimated using Poisson pseudo-maximum likelihood (PPML) with population as the exposure variable. Robust standard errors are reported in parentheses and clustered at the city level. All models include City × Year and Year × Quarter fixed effects. $p < 0.10$, $p < 0.05$, $p < 0.01$.

Table S13. Formal Tests of Heterogeneity in Power Outage Effects on Mental Health Hospitalizations, Related to Figure 4

Dimension	Classification	Group 1	IRR	p-value	Group 2	IRR	p-value	Z-test p-value	Interaction p-value
Urban/Rural	RUCC=1 vs Rest	Large Metro	1.016	0.024**	Non-Large Metro	0.973	0.086*	0.013**	0.015**
Poverty	Census ≥20%	Low Poverty	1.015	0.031**	High Poverty	1.032	0.183	0.499	0.084*
Medicaid	≥25% threshold	Low Medicaid	1.008	0.407	High Medicaid	1.022	0.020**	0.313	0.483
Season	Warm/Cold	Cold Season	1.007	0.445	Warm Season	1.020	0.056*	0.353	0.223
Gender	Median split	Low Male %	1.008	0.311	High Male %	1.023	0.057*	0.297	0.907

Note: Each row reports split-sample PPML-HDFE regression results for the outage variable (ln_outage_1hr_p75). IRR = incidence rate ratio. Z-test p-values are computed as $z = (b_1 - b_2) / \sqrt{SE_1^2 + SE_2^2}$. Interaction p-values are from models including the outage × subgroup interaction term. Urban–rural status is classified using USDA Rural-Urban Continuum Codes (RUCC = 1 for large metropolitan areas with ≥1 million population). High Poverty is defined using the U.S. Census Bureau threshold of ≥20% poverty rate. High Medicaid is defined as ZIP codes where ≥25% of hospitalized patients are covered by Medicaid. Warm Season includes Q2–Q3 (April–September); Cold Season includes Q1 and Q4. Gender composition is based on a median split of the ZIP code–level share of male patients. All models include ZIP code and year × quarter fixed effects with total population as exposure. Standard errors clustered at the ZIP code level. * $p < 0.10$, ** $p < 0.05$.

Table S14. Robustness Check – Raw Outage Episode Count as Alternative Exposure Measure, Related to Figure 2

Model	(1)	(2)
	Baseline	Raw Count
Dependent Variables		
ln_outage_1hr_p75	1.015**	
	(0.008)	
new_1hr_episodes_p75		1.002*
		(0.001)
extreme_heat_days_p90	1.002**	1.002**
	(0.001)	(0.001)
avg_cmr_clean	1.050***	1.050***
	(0.008)	(0.008)
avg_cmr_mortality_clean	0.943***	0.943***
	(0.005)	(0.005)
poverty_rate	1.008***	1.008***
	(0.003)	(0.003)
pct_male	1.006***	1.006***
	(0.001)	(0.001)
median_income_1k	1.002*	1.002*
	(0.001)	(0.001)
black_pct	0.970	0.965
	(0.293)	(0.291)
asian_pct	0.331**	0.331**
	(0.157)	(0.157)
pct_medicaid	1.010***	1.010***
	(0.001)	(0.001)
pct_uninsured	1.009***	1.009***
	(0.003)	(0.003)
psych_100k	1.025***	1.025***
	(0.005)	(0.005)
Observations	8,152	8,152
ZIP FE	YES	YES
Year*Quarter FE	YES	YES
Exposure	Population	Population

Note: The dependent variable is the number of hospitalizations with a primary mental health diagnosis. Models are estimated using Poisson pseudo-maximum likelihood (PPML) with population as the exposure variable. Robust standard errors are reported in parentheses and clustered at the city level. All models include City × Year and Year × Quarter fixed effects. $p < 0.10$, p < 0.05, $p < 0.01$.

Table S15. Robustness – power outage effect under each extreme-heat threshold within the same specification, Related to Figure 2

	**(1)**	**(2)**	**(3)**	**(4)**	**(5)**	**(6)**

	Model 3 controls Heat ⩾ P75	Model 3 controls Heat ⩾ P90	Model 3 controls Heat ⩾ P95	Model 5 controls Heat ⩾ P75	Model 5 controls Heat ⩾ P90	Model 5 controls Heat ⩾ P95
**ln(outage episodes + 1)**	1.016**<br>(0.008)	1.015**<br>(0.008)	1.015**<br>(0.008)	1.013*<br>(0.007)	1.013*<br>(0.007)	1.013*<br>(0.007)
**Extreme heat days (⩾ P75)**	1.001<br>(0.001)			1.000<br>(0.001)		
**Extreme heat days (⩾ P90)**		1.002**<br>(0.001)			1.002**<br>(0.001)	
**Extreme heat days (⩾ P95)**			1.003***<br>(0.001)			1.003***<br>(0.001)
**Observations**	8,152	8,152	8,152	8,152	8,152	8,152
**Clusters**	430	430	430	430	430	430
**Pseudo R^2**	0.866	0.866	0.866	0.868	0.868	0.868

Notes: Exponentiated coefficients (incidence rate ratios); standard errors clustered at the ZIP code level in parentheses. * $p < 0.10$, ** $p < 0.05$, *** $p < 0.01$. All models include ZIP code and Year × Quarter fixed effects, with total population as the exposure term. Each column keeps the control variables of the indicated Figure 1 model and changes only the extreme-heat threshold: columns (1)–(3) use the Model 3 (main specification) controls with heat defined at the 75th, 90th, and 95th percentiles, respectively; columns (4)–(6) repeat this for the Model 5 (disease-specific comorbidity) controls. The power outage coefficient is unchanged to the third decimal across heat thresholds within each set of controls, while extreme heat is significant only at the 90th and 95th percentile thresholds.

Table S16. Power outage effects by diagnostic position of mental health (PPML-HDFE), Related to Figure 1

	(1) Primary mental health	(2) Mental health as secondary diagnosis	(3) No mental health code (contrast)
**ln(outage episodes + 1)**	1.013*<br>(0.007)	0.996<br>(0.004)	1.007**<br>(0.003)
**Extreme heat days (⩾ P90)**	1.002**<br>(0.001)	1.001**<br>(0.000)	1.001*<br>(0.000)
**Observations**	8,533	8,573	8,573
**Clusters**	435	439	439
**Pseudo R^2**	0.868	0.958	0.966

Notes: Exponentiated coefficients (incidence rate ratios); standard errors clustered at the ZIP code level in parentheses. * $p < 0.10$, ** $p < 0.05$, *** $p < 0.01$. All models include ZIP code and Year × Quarter fixed effects with total population as the exposure term, and the Model 3 controls excluding the two Elixhauser comorbidity indices, which are constructed from the same secondary diagnosis fields as the outcomes in columns (2)–(3). Column (1) counts admissions with a primary mental health diagnosis; column (2) counts admissions with a non-mental-health principal diagnosis and at least one mental health code in the secondary diagnosis fields; column (3) counts admissions with no mental health code in any position. Results with the full Model 3 control set are similar (column 1: IRR = 1.015, $p < 0.05$; column 2: IRR = 0.999; column 3: IRR = 1.004, not significant). The null result in column (2) is unchanged across 24 alternative outage definitions (duration thresholds of 1–8 hours × 50th/75th/90th severity percentiles; IRRs range from 0.996 to 1.001, none significant).

Table S17. Power outage effects in vulnerable populations, by coexisting depression or anxiety (PPML-HDFE), Related to Figure 4

	Diabetes +D/A	Diabetes −D/A	COPD/Asthma +D/A	COPD/Asthma −D/A	Heart disease +D/A	Heart disease −D/A	Dementia +D/A
**ln(outage episodes + 1)**	1.025 (0.025)	1.010 (0.013)	0.996 (0.021)	1.000 (0.014)	0.989 (0.022)	0.999 (0.012)	1.075 (0.085)
**Observations**	7,406	8,182	7,742	8,104	7,714	8,466	4,619
**Clusters**	367	409	386	405	382	428	231

Notes: Exponentiated coefficients (incidence rate ratios); standard errors clustered at the ZIP code level in parentheses. * $p < 0.10$, ** $p < 0.05$, *** $p < 0.01$. Each column uses the main specification excluding the two Elixhauser comorbidity indices (see Note S4). Vulnerable groups are defined by the principal diagnosis (diabetes E08–E13; COPD/asthma J40–J47; heart disease I20–I25 and I50; dementia G30–G31; chronic kidney disease N18); +D/A columns count admissions with coexisting depression or anxiety (F32, F33, F41, F43) in the secondary diagnosis fields, −D/A columns count the remainder. The dementia and chronic kidney disease groups are small (3,993 and 541 admissions statewide), so these estimates—including the significant estimate for chronic kidney disease—should be treated as suggestive.

Supplemental references

[S1] Do, V., McBrien, H., Flores, N.M., Northrop, A.J., Schlegelmilch, J., Kiang, M.V., and Casey, J.A. (2023). Spatiotemporal distribution of power outages with climate events and social vulnerability in the USA. Nat Commun *14*, 2470. https://doi.org/10.1038/s41467-023-38084-6.

[S2] Do, V., Wilner, L.B., Flores, N.M., McBrien, H., Northrop, A.J., and Casey, J.A. (2025). Spatiotemporal patterns of individual and multiple simultaneous severe weather events co-occurring with power outages in the United States, 2018–2020. PLOS Clim *4*, e0000523. https://doi.org/10.1371/journal.pclm.0000523.

[S3] Bourque, F., and Cunsolo Willox, A. (2014). Climate change: The next challenge for public mental health? International Review of Psychiatry *26*, 415–422. https://doi.org/10.3109/09540261.2014.925851.

[S4] Clayton, S., Devine-Wright, P., Stern, P.C., Whitmarsh, L., Carrico, A., Steg, L., Swim, J., and Bonnes, M. (2015). Psychological research and global climate change. Nature Clim Change *5*, 640–646. https://doi.org/10.1038/nclimate2622.

[S5] Palinkas, L.A., and Wong, M. (2020). Global climate change and mental health. Current Opinion in Psychology *32*, 12–16. https://doi.org/10.1016/j.copsyc.2019.06.023.

[S6] Baecker, L., Iyengar, U., Del Piccolo, M.C., and Mechelli, A. (2025). Impacts of extreme heat on mental health: Systematic review and qualitative investigation of the underpinning mechanisms. The Journal of Climate Change and Health *22*, 100446. https://doi.org/10.1016/j.joclim.2025.100446.

[S7] Basu, R., Gavin, L., Pearson, D., Ebisu, K., and Malig, B. (2018). Examining the Association Between Apparent Temperature and Mental Health-Related Emergency Room Visits in California. American Journal of Epidemiology *187*, 726–735. https://doi.org/10.1093/aje/kwx295.

[S8] Li, D., Zhang, Y., Li, X., Zhang, K., Lu, Y., and Brown, R.D. (2023). Climatic and meteorological exposure and mental and behavioral health: A systematic review and meta-analysis. Science of The Total Environment *892*, 164435. https://doi.org/10.1016/j.scitotenv.2023.164435.

[S9] Rony, M.K.K., and Alamgir, H.M. (2023). High temperatures on mental health: Recognizing the association and the need for proactive strategies—A perspective. Health Science Reports *6*, e1729. https://doi.org/10.1002/hsr2.1729.

[S10] Lõhmus, M. (2018). Possible Biological Mechanisms Linking Mental Health and Heat—A Contemplative Review. IJERPH *15*, 1515. https://doi.org/10.3390/ijerph15071515.

[S11] Page, L.A., Hajat, S., Kovats, R.S., and Howard, L.M. (2012). Temperature-related deaths in people with psychosis, dementia and substance misuse. Br J Psychiatry *200*, 485–490. https://doi.org/10.1192/bjp.bp.111.100404.

[S12] Meadows, J., Mansour, A., Gatto, M.R., Li, A., Howard, A., and Bentley, R. (2024). Mental illness and increased vulnerability to negative health effects from extreme heat events: a systematic review. Psychiatry Research *332*, 115678. https://doi.org/10.1016/j.psychres.2023.115678.

[S13] Casey, J.A., Fukurai, M., Hernández, D., Balsari, S., and Kiang, M.V. (2020). Power Outages and Community Health: a Narrative Review. Curr Envir Health Rpt *7*, 371–383. https://doi.org/10.1007/s40572-020-00295-0.

[S14] Klinger, C., Landeg, O., and Murray, V. (2014). Power Outages, Extreme Events and Health: a Systematic Review of the Literature from 2011-2012. PLoS Curr. https://doi.org/10.1371/currents.dis.04eb1dc5e73dd1377e05a10e9edde673.

[S15] Rubin, G.J., and Rogers, M.B. (2019). Behavioural and psychological responses of the public during a major power outage: A literature review. International Journal of Disaster Risk Reduction *38*, 101226. https://doi.org/10.1016/j.ijdrr.2019.101226.

[S16] Grineski, S., Scott, M., Collins, T., Chakraborty, J., and Ramos, K. (2023). Anxiety and depression after winter storm Uri: Cascading disasters and mental health inequities. International Journal of Disaster Risk Reduction *96*, 103933. https://doi.org/10.1016/j.ijdrr.2023.103933.

[S17] Sugg, M.M., Wertis, L., Ryan, S.C., Green, S., Singh, D., and Runkle, J.D. (2023). Cascading disasters and mental health: The February 2021 winter storm and power crisis in Texas, USA. Science of The Total Environment *880*, 163231. https://doi.org/10.1016/j.scitotenv.2023.163231.

[S18] Casey, J.A., Mango, M., Mullendore, S., Kiang, M.V., Hernández, D., Li, B.H., Li, K., Im, T.M., and Tartof, S.Y. (2021). Trends from 2008 to 2018 in Electricity-dependent Durable Medical Equipment Rentals and Sociodemographic Disparities. Epidemiology *32*, 327–335. https://doi.org/10.1097/EDE.0000000000001333.

[S19] Bhattacharyya, A., and Hastak, M. (2023). A data-driven approach to quantify disparities in power outages. Sci Rep *13*, 7247. https://doi.org/10.1038/s41598-023-34186-9.

[S20] Andresen, A.X., Kurtz, L.C., Hondula, D.M., Meerow, S., and Gall, M. (2023). Understanding the social impacts of power outages in North America: a systematic review. Environ. Res. Lett. *18*, 053004. https://doi.org/10.1088/1748-9326/acc7b9.

[S21] Dominianni, C., Lane, K., Johnson, S., Ito, K., and Matte, T. (2018). Health Impacts of Citywide and Localized Power Outages in New York City. Environ Health Perspect *126*, 067003. https://doi.org/10.1289/EHP2154.

[S22] Cook, B.L., Zuvekas, S.H., Chen, J., Progovac, A., and Lincoln, A.K. (2017). Assessing the Individual, Neighborhood, and Policy Predictors of Disparities in Mental Health Care. Med Care Res Rev *74*, 404–430. https://doi.org/10.1177/1077558716646898.

[S23] Gibbons, R.D., Olfson, M., Saulsberry, L., Edlund, M.J., Zangeneh, S., Bareis, N., Chwastiak, L., Gibbons, J.B., and Kessler, R.C. (2024). Social Vulnerability and Prevalence and Treatment for Mental Health and Substance Use Disorders. JAMA Psychiatry *81*, 976. https://doi.org/10.1001/jamapsychiatry.2024.1870.

[S24] Buchner, M., and Rehm, M. (2025). Energy poverty and health: Micro-level evidence from Germany. Energy Economics *145*, 108376. https://doi.org/10.1016/j.eneco.2025.108376.

[S25] Lin, S., Lu, Y., Justino, J., Dong, G., and Lauper, U. (2016). What Happened to Our Environment and Mental Health as a Result of Hurricane Sandy? Disaster med. public health prep. *10*, 314–319. https://doi.org/10.1017/dmp.2016.51.

[S26] Grineski, S.E., Collins, T.W., and Chakraborty, J. (2022). Cascading disasters and mental health inequities: Winter Storm Uri, COVID-19 and post-traumatic stress in Texas. Social Science & Medicine *315*, 115523. https://doi.org/10.1016/j.socscimed.2022.115523.

[S27] Sheridan, S.C., Zhang, W., Deng, X., and Lin, S. (2021). The individual and synergistic impacts of windstorms and power outages on injury ED visits in New York State. Science of The Total Environment *797*, 149199. https://doi.org/10.1016/j.scitotenv.2021.149199.

[S28] Skarha, J., Gordon, L., Sakib, N., June, J., Jester, D.J., Peterson, L.J., Andel, R., and Dosa, D.M. (2021). Association of Power Outage With Mortality and Hospitalizations Among Florida Nursing Home Residents After Hurricane Irma. JAMA Health Forum *2*, e213900. https://doi.org/10.1001/jamahealthforum.2021.3900.

[S29] Lin, S., Zhang, W., Sheridan, S., Mongillo, M., DiRienzo, S., Stuart, N.A., Stern, E.K., Birkhead, G., Dong, G., Wu, S., et al. (2021). The immediate effects of winter storms and power outages on multiple health outcomes and the time windows of vulnerability. Environmental Research *196*, 110924. https://doi.org/10.1016/j.envres.2021.110924.

[S30] Sovacool, B.K., Carley, S., and Kiesling, L. (2024). Energy justice beyond the wire: Exploring the multidimensional inequities of the electrical power grid in the United States. Energy Research & Social Science *111*, 103474. https://doi.org/10.1016/j.erss.2024.103474.

[S31] ICD-10-CM/PCS Resources (2022). Healthcare Cost and Utilization Project (HCUP). https://www.hcup-us.ahrq.gov/datainnovations/icd10_resources.jsp.

[S32] PowerOutage.com Live US Power Outages. https://poweroutage.us/.

[S33] Richards, C.A., Amiri, S., Walden, V.P., Postma, J., Heidari Kapourchali, M., and Zuur, A.F. (2024). Association of social vulnerability factors with power outage burden in Washington state: 2018–2021. PLoS ONE *19*, e0307742. https://doi.org/10.1371/journal.pone.0307742.

[S34] Wang, R., Vanga, V.L.M., Zaiken, Z.B., and Bennett, J. (2022). Analysis of the electric power outage data and prediction of electric power outage for major metropolitan areas in Texas using Machine Learning and Time Series Methods. SMU Data Science Review *6*.

[S35] Deng, X., Friedman, S., Ryan, I., Zhang, W., Dong, G., Rodriguez, H., Yu, F., Huang, W., Nair, A., Luo, G., et al. (2022). The independent and synergistic impacts of power outages and floods on hospital admissions for multiple diseases. Science of The Total Environment *828*, 154305. https://doi.org/10.1016/j.scitotenv.2022.154305.

[S36] Zhang, W., Sheridan, S.C., Birkhead, G.S., Croft, D.P., Brotzge, J.A., Justino, J.G., Stuart, N.A., Du, Z., Romeiko, X.X., Ye, B., et al. (2020). Power Outage An Ignored Risk Factor for COPD Exacerbations. Chest *158*, 2346–2357. https://doi.org/10.1016/j.chest.2020.05.555.

[S37] Kaiser, R., Rubin, C.H., Henderson, A.K., Wolfe, M.I., Kieszak, S., Parrott, C.L., and Adcock, M. (2001). Heat-Related Death and Mental Illness During the 1999 Cincinnati Heat Wave: The American Journal of Forensic Medicine and Pathology *22*, 303–307. https://doi.org/10.1097/00000433-200109000-00022.
